# Strecker degradation of amino acids promoted by a camphor-derived sulfonamide

**DOI:** 10.3762/bjoc.12.73

**Published:** 2016-04-18

**Authors:** M Fernanda N N Carvalho, M João Ferreira, Ana S O Knittel, Maria da Conceição Oliveira, João Costa Pessoa, Rudolf Herrmann, Gabriele Wagner

**Affiliations:** 1 Centro de Química Estrutural, Instituto Superior Técnico, Universidade de Lisboa, Av. Rovisco Pais 1049-001 Lisboa, Portugal; 2 Institut für Physik, Universität Augsburg, Universitätsstr. 2, 86159 Augsburg, Germany; 3 University of Chester, Faculty of Science and Engineering, Department of Natural Sciences, Thornton Science Park, Pool Lane, Ince, Chester, CH2 4NU, UK

**Keywords:** amino acids, camphorsulfonylimine, DFT calculations, NMR characterization, Strecker degradation

## Abstract

A camphor-derived sulfonimine with a conjugated carbonyl group, oxoimine **1** (O_2_SNC_10_H_13_O), reacts with amino acids (glycine, L-alanine, L-phenylalanine, L-leucine) to form a compound O_2_SNC_10_H_13_NC_10_H_14_NSO_2_ (**2**) which was characterized by spectroscopic means (MS and NMR) and supported by DFT calculations. The product, a single diastereoisomer, contains two oxoimine units connected by a –N= bridge, and thus has a structural analogy to the colored product Ruhemann´s purple obtained by the ninhydrin reaction with amino acids. A plausible reaction mechanism that involves zwitterions, a Strecker degradation of an intermediate imine and water-catalyzed tautomerizations was developed by means of DFT calculations on potential transition states.

## Introduction

**Figure 1:**
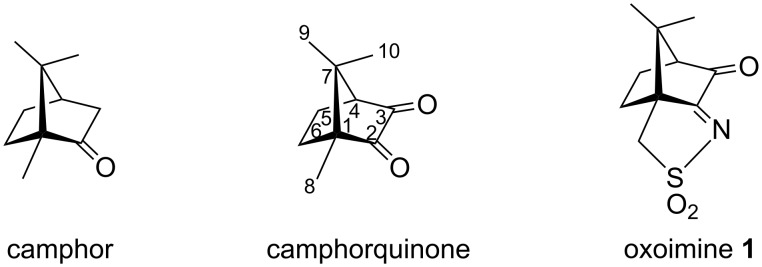
Camphor and some camphor derivatives.

Among the many derivatives of natural camphor, oxoimine **1** (Figure 1) shows an especially versatile chemistry. The C=O double bond can be converted to ketals, thioketals or even dihalogenomethylenes [1] or react to form hydrazones [2]. On the other hand the C=N double bond can be converted into an oxaziridine group forming chiral oxaziridines which act as enantioselective oxidation reagents [1,3–5]. Other types of reactions include the alkylation of the imine nitrogen atom followed by ring annulation [6], cleavage of the sulfonimine [7–8] or camphor [9] rings, reduction of the CO or CN double bonds [10], addition of acetylide anions to form dialkynes that can undergo complex skeletal rearrangements and unusual redox reactions [11–14].

**Scheme 1:**
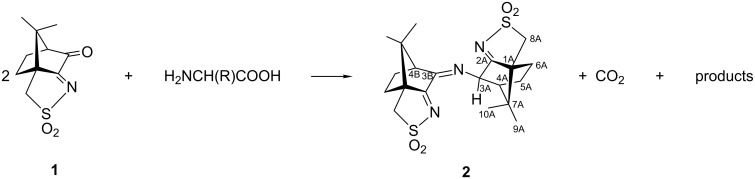
Formation of **2** from reaction of oxoimine **1** with amino acids (H_2_NCH(R)COOH: R = H, CH_3_, CH_2_Ph, CH_2_CH(CH_3_)_2_) and carbon atoms numbering for **2**.

Since camphorquinone (Figure 1) is known to react with amines through the C=O group (position 3, Figure 1) forming imine camphor compounds [2,15–17], one could expect that an amine condensation with the C=O double bond of oxoimine **1** would also lead to imine or aminal (carbinolamine)-type compounds. Thus, we set out to investigate the reaction of **1** with amino acids. Simple imines could not be observed. Instead, compound **2** was obtained (Scheme 1), containing two camphor-derived moieties, bridged by a nitrogen atom, and its structure was fully elucidated by spectroscopic means. Surprisingly, in **2** the only obvious trace of the amino acid is the bridging nitrogen atom, which means that the rest of the amino acid disappeared.

From reactions of **1** with several amino acids (glycine, alanine, leucine, phenylalanine) always compound **2** was obtained. This observation suggests a reaction similar to the “ninhydrin reaction” which is used in the identification/analysis of amino acids [18]. The central part of this reaction is the Strecker degradation [19] of an intermediate imine. The surprising instability of the otherwise robust amino acids upon the formation of imines with certain carbonyl compounds is the basis for enzymatic transamination and many other reactions [20–21] while the Strecker degradation is one cornerstone in food chemistry [22].

We present here details on the characterization of compound **2**, which extends the scope of Strecker degradation of amino acids to reactions with oxo-sulfonimine compounds and investigate the reaction mechanism of its formation by computational means.

## Results and Discussion

### Synthesis and characterization

The α-amino acids (H_2_NCHRCOOH) react with 3-oxocamphorsulfonylimine (O_2_SNC_10_H_13_O, **1**, oxoimine) forming a pale yellow solid (**2**) that unexpectedly displays analytical and spectroscopic data (NMR, IR) independent of the amino acid (R = H, glycine; R = CH_3_, L-alanine; R = CH_2_Ph, L-phenylalanine; R = CH_2_CH(CH_3_)_2_, leucine).

The absence of the characteristic oxoimine CO stretching band (1760 cm^−1^) in the IR spectrum of **2** (see Supporting Information File 1, Figure S1), shows that the reaction occurs at position 3 of the camphor skeleton (Scheme 1, for labeling). In addition, the observation of partially overlapping IR bands (ν_CN_, 1676, 1641 cm^−1^) indicate distinct imine groups in agreement with the signals observed in the ^13^C NMR spectrum at 194.8, 186.4 ppm (sulfonimine) and 173.3 ppm (camphorimine). See Supporting Information File 1 for experimental and Supporting Information File 2 for calculated NMR spectra. This unexpected compound was formulated as O_2_SNC_10_H_13_NC_10_H_14_NSO_2_ (Scheme 1) based on elemental analysis and ESIMS data. The elemental analysis is consistent with three nitrogen atoms per molecule in **2** and high resolution ESIMS analysis and tandem mass spectrometry experiments further corroborate the formulation.

**Figure 2:**
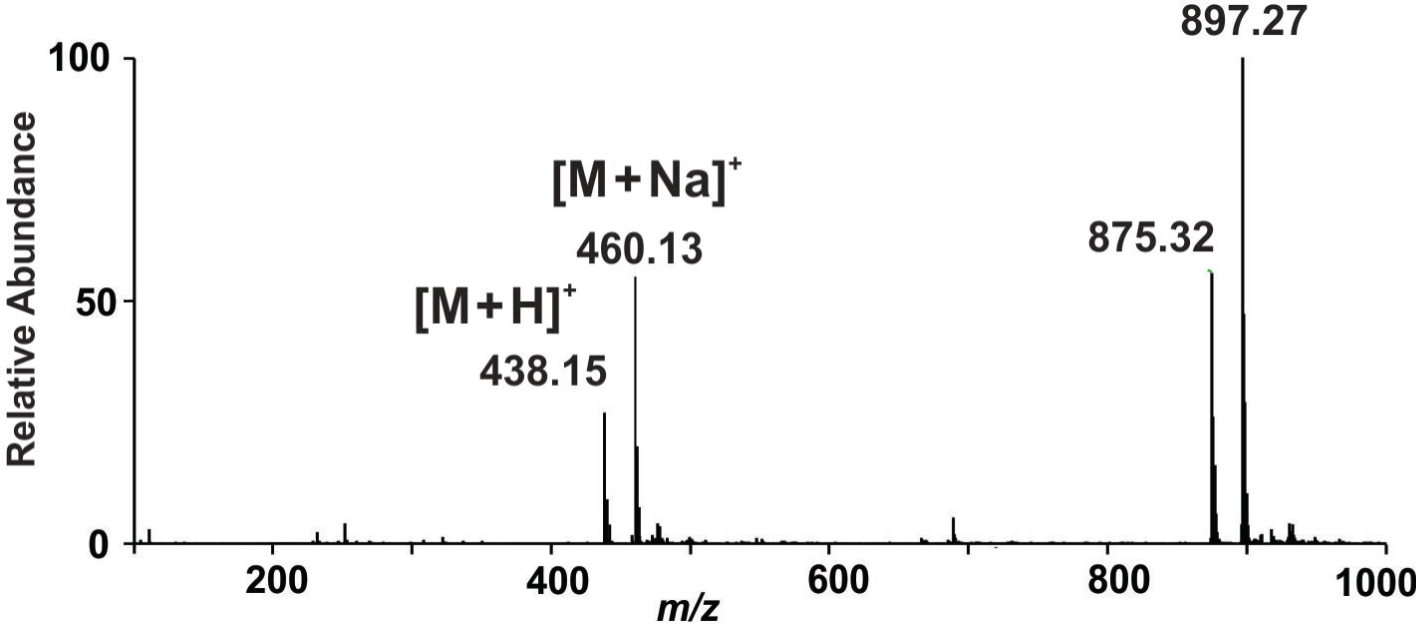
ESI mass spectrum of **2** (positive ion mode).

The positive-ion (+)ESIMS of **2** in acetonitrile shows two sets of peaks at *m*/*z* 438 and 460 assigned to the protonated molecule [M + H]^+^ and its sodium adduct [M + Na]^+^, respectively (Figure 2). The main set of peaks at *m*/*z* 875 and 897 are attributed to the dimeric species, [2M + H]^+^ and [2M + Na]^+^, respectively, formed in the ion phase. The even nominal mass found for the protonated molecule dictates, according to the nitrogen rule [23], an odd number of nitrogen atoms in the structure of **2**, that was further corroborated by collision-induced dissociation experiments. The tandem mass spectrum of the protonated molecule of **2** can be found in Supporting Information File 1, Figure S7.

The major fragmentation pathway is due to the loss of 64 Da (SO_2_) leading to an ion at an even value (*m*/*z* 374). The sequential loss of sulfur dioxide plus ammonia gives a fragment ion with *m*/*z* 357, indicating an even number of nitrogen atoms. Further loss of a camphor moiety (C_10_H_15_NSO_2_) displays an ion at *m*/*z* 227 supporting a species having an even number of nitrogen atoms (see Supporting Information File 1, Figure S7 showing a scheme with the fragmentation mechanism of **2**). The formulation of **2** was corroborated by accurate mass measurements using QqTOF-MS which led to the following results (M = measured monoisotopic mass of precursor ion; C = calculated monoisotopic mass of precursor ion; Δ = deviation (ppm); e− = electron configuration; NR = nitrogen rule; F = deduced formula for precursor ion): M = 438.1506; C = 438.1516; Δ = −2.1; e− = even; NR = ok; F = C_20_H_28_N_3_O_4_S_2_.

Whereas no doubts exist concerning the analytical formulation of **2**, its structure remained unclear. In the absence of crystals suitable for single crystal X-ray diffraction analysis, this point was fully clarified by NMR (Supporting Information File 1, Figure S2–S6) and further supported by calculations.

**Figure 3:**
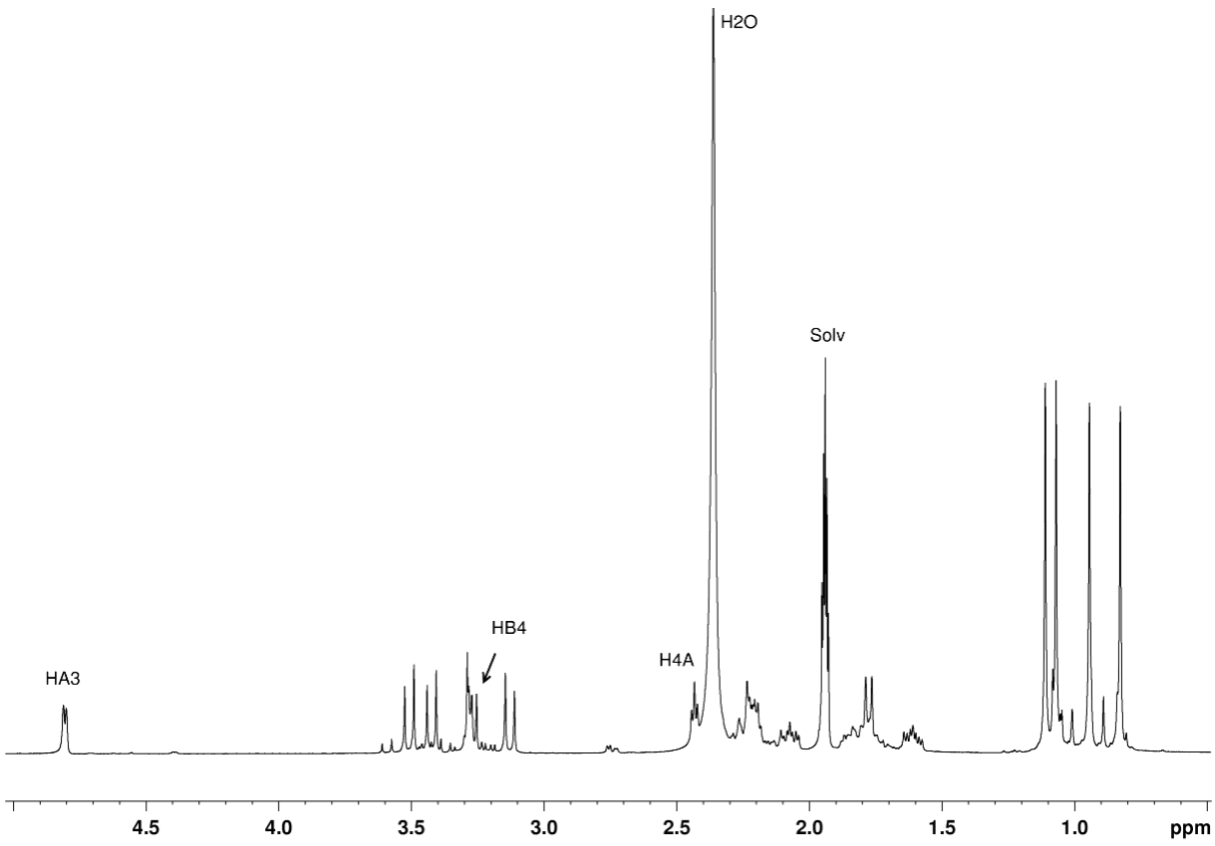
^1^H NMR spectrum of **2** in CD_3_CN at *T* = −20 °C.

The assignment of all resonances in the ^1^H and ^13^C NMR spectra including *endo* and *exo* atoms of the methylene groups (CH_2_) was accomplished by combining ^1^H, ^13^C, DEPT and 2D NMR data (HSQC, HMBC and NOESY). In the ^1^H NMR spectrum (Figure 3) two sets of two signals of equal intensity in the range of 1.2 to 0.80 ppm are consistent with two pairs of non-equivalent methyl groups. One doublet (4.81 ppm, H_A_-3) and one triplet (2.43 ppm, H_A_-4) of equal integration (1H), in addition to three sets of multiplets with chemical shifts in the range usually assigned to the methylene groups (5A, 6A) evidence the camphor unit A (see Scheme 1 for atom numbering).

The proton H4B appears as doublet due to the coupling with H5B(exo) while the corresponding coupling constant to H5B(endo) is predicted from the Karplus equation to be low (0.2 Hz) according to the calculated dihedral angle H4B–C4B–C5B–H5B(endo) of 76.1°, and is thus not resolved and leads only to a broadening of the signal. Analogously, for proton H4A a triplet is observed due to the additional coupling to proton H3A, with a dihedral angle H4A–C4A–C5A–H5A(endo) of 77.7° corresponding to a coupling constant of 0.1 Hz.

**Figure 4:**
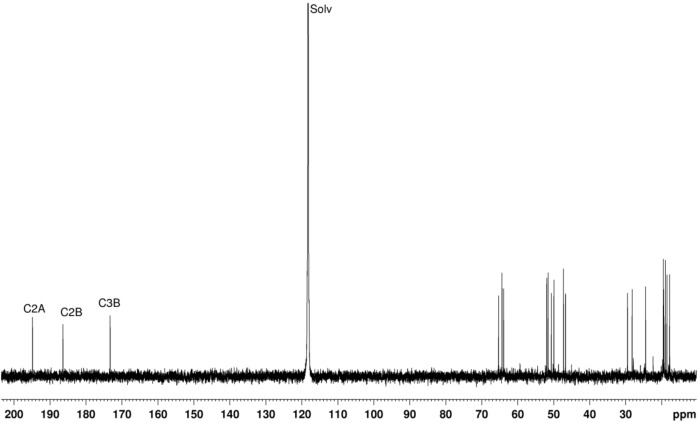
^13^C NMR spectrum of **2** in CD_3_CN at *T* = −20 °C.

The two camphor sulfonylimine fragments are positioned in such a way that H4B (3.28 ppm) gives NOE with H4A (2.43 ppm) and H3A (4.81 ppm) (Supporting Information File 1). The ^1^H NMR spectrum further supports two distinct oxoimine units in the same molecule. No N–H signals were detected conceivably due to traces of water that promotes N–H fast (on the NMR timescale) proton exchange. Three downfield signals observed in the ^13^C NMR spectrum (Figure 4) indicate distinct carbon–nitrogen double bonds, in agreement with the IR data. Cross peaks between H3A (4.81 ppm) and C3B (173.3 ppm), C2A (194.9 ppm), 19.4 (C5A) observed in the HMBC spectrum support bridging of the two camphor sulfonylimine units bound by nitrogen at C3 (Supporting Information File 1, Figure S4).

The structure of compound **2** thus resembles very much that of Ruhemann’s purple [24], the compound responsible for the intense color developed in the reaction of ninhydrin with amino acids. Compound **2** is not intensely colored, since its carbon–nitrogen double bonds are not connected to an aromatic system and therefore electron density cannot be delocalized. Having established the overall dimeric structure of **2**, next the configuration at the newly formed chiral center at carbon atom 3A has to be determined.

### Calculations

#### Structure optimization

**Figure 5:**
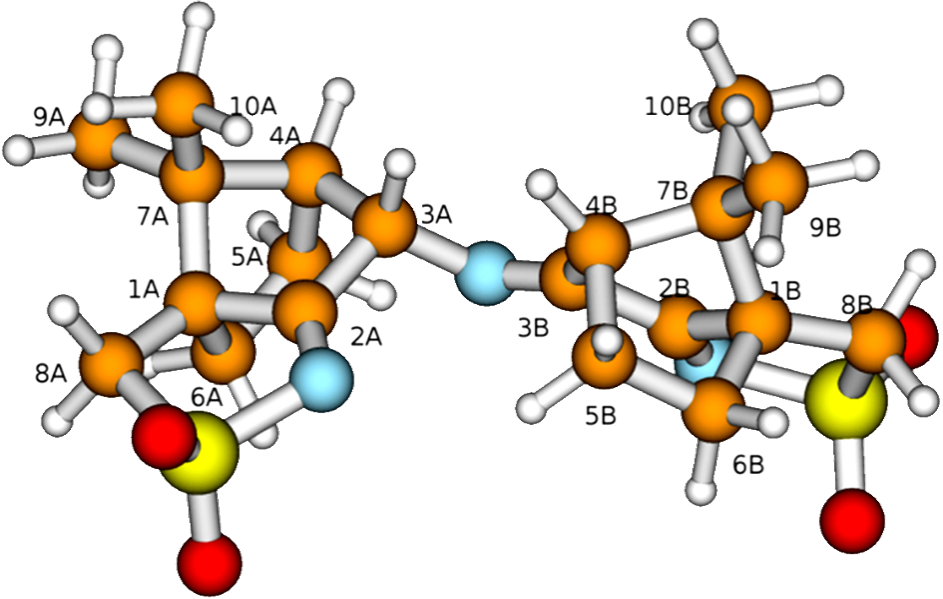
Optimized structure of **2** ((*S*)-3A isomer) with labeling scheme.

To clarify this point, we have calculated the optimized structures of both isomers with H3A in *endo* position ((*R*)-3A) and H3A in *exo* position ((*S*)-3A) which are almost equal in energy; the (*S*)-3A isomer being slightly more stable (0.4 kcal/mol) than the (*R*)-3A isomer. The optimized structure of the (*S*)-3A isomer is displayed in Figure 5.

**Figure 6:**
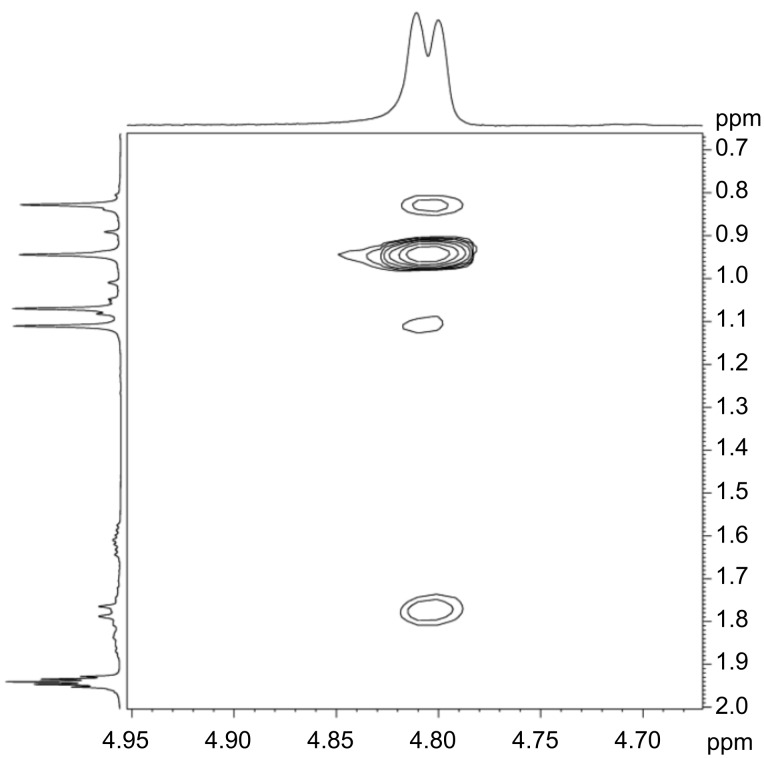
NOESY spectrum (detail) showing the cross peak between H3A and H10A (see Supporting Information File 1, Figure S6 for the full spectrum).

Assuming that to observe NOE effects in the 2D NMR spectra a distance lower than ca. 3 Å between the hydrogen atoms is required, the calculated structures indicate that H3A should show a strong NOE to H10A (one of the methyl groups) only in the isomer with H3A in *exo* position, i.e., (*S*)-3A isomer (distance 2.177 Å), while the isomer (*R*)-3A (with H3A in *endo* position) should show NOEs of H3A to H5A *endo* (distance 2.243 Å) and H5B *endo* (distance 2.991 Å). Based on the calculations, no other significant differences between (*S*)-3A and (*R*)-3A isomers are expected; the calculated distance of H3A to H4B in the second camphor moiety is 2.181 Å in the isomer with (*S*)-3A and 2.459 Å in (*R*)-3A, i.e., they do not differ sufficiently to allow for a decision between the isomers on the basis of NOE effects. Thus, the strong cross peak between H10A and H3A observed in the experimental NOESY spectrum (Figure 6) allows the assignment of the configuration (*S*)-3A (with H3A in *exo* position) to compound **2**.

The attribution was further confirmed by comparing the calculated chemical shifts for all carbon and hydrogen atoms with the experimental data obtained for compound **2** (see Supporting Information File 2 for calculations). In the ^1^H NMR spectrum a coupling constant of 4.4 Hz was measured for H3A and H4A coupling. From the calculated structures of both isomers of **2**, dihedral angles between these two atoms are 48.8° and 86.7°, respectively, for the (*S*)-3A and the (*R*)-3A isomer. According to the Karplus equation, these values correspond to coupling constants of 5.0–5.5 Hz and 1.5–2.0 Hz, respectively, thus corroborating the (*S*)-3A configurational assignment.

#### Mechanism

Having identified and structurally characterized compound **2** it remained to explain how it formed under the experimental conditions and whether the preferential formation of the isomer with *S*-configuration at 3A can be explained. In water/ethanol the amino acids and the oxoimine **1** are moderately soluble. Acetic acid keeps the pH of the mixture in the range 4–5, where the acid-catalyzed imine formation from amino groups and carbonyl compounds as well as imine hydrolysis can easily occur [25].

The key step in the reaction sequence leading to compound **2**, would then be the loss of CO_2_ by a variant of the Strecker degradation. This step is taken “as granted” and is not further questioned in most investigations, the focus being in the tautomerization reactions (H migrations) occurring later in the sequence. To get an insight into the process we carried out calculations on the Strecker degradation of glycine induced by glyoxal or ninhydrin and the results are presented in Supporting Information File 3. From these calculations we can draw the following conclusions: i) imines formed by reaction of amino acids with carbonyl compounds have the tendency to lose CO_2_ upon deprotonation of the carboxyl group. Without a negative charge at the carboxyl group, decarboxylation is difficult; ii) whether the deprotonation is followed by a decarboxylation or not, depends on the ability of the remaining molecule to accommodate the negative charge; iii) the best way to achieve this accommodation is by “delocalization” over one or more double bonds. Because of this, imines of 1,2-dicarbonyl compounds (like glyoxal and ninhydrin) are particularly well-suited for decarboxylation. When such an imine can establish a zwitterionic structure with a deprotonated carboxyl and a protonated carbonyl oxygen or imine nitrogen, decarboxylation is fast and efficient. When trying to optimize the structure of such zwitterions, decarboxylation occurs instead. Thus, we do not find a transition state, and the activation barrier approaches zero. Experimentally, Strecker degradations are not instantaneous, which means that there must exist at least a small barrier somewhere in the reaction sequence. One may expect such a barrier in the tautomerization (probably catalyzed by water) which forms the zwitterion by proton migration from the neutral imine.

The experimental results on Strecker degradation have been summarized in a rule that says that the most efficient promoters have a structure of the type O=C–(CH=CH)*_n_*–C=O (*n* = 0, 1, ...) [26–27]. We now assume that the same is true if one C=O group is replaced by one C=NR (R is a sulfonyl group) as in the case of oxoimine **1**. In this way, it would be expected that the reactivity is reduced due to the electron-withdrawing effect of the sulfonyl group which makes protonation of the nitrogen atom more difficult than a R = alkyl or aryl group. Such protonation is necessary to form a zwitterionic structure of the type discussed above. Despite this effect, the Strecker degradation of the amino acids glycine, alanine, leucine and phenylalanine induced by **1** proceeds rapidly under mild conditions. The only other camphor derivative found to promote the Strecker degradation is camphorquinone [27] (Figure 1), no Strecker degradation is observed with camphor itself.

**Figure 7:**
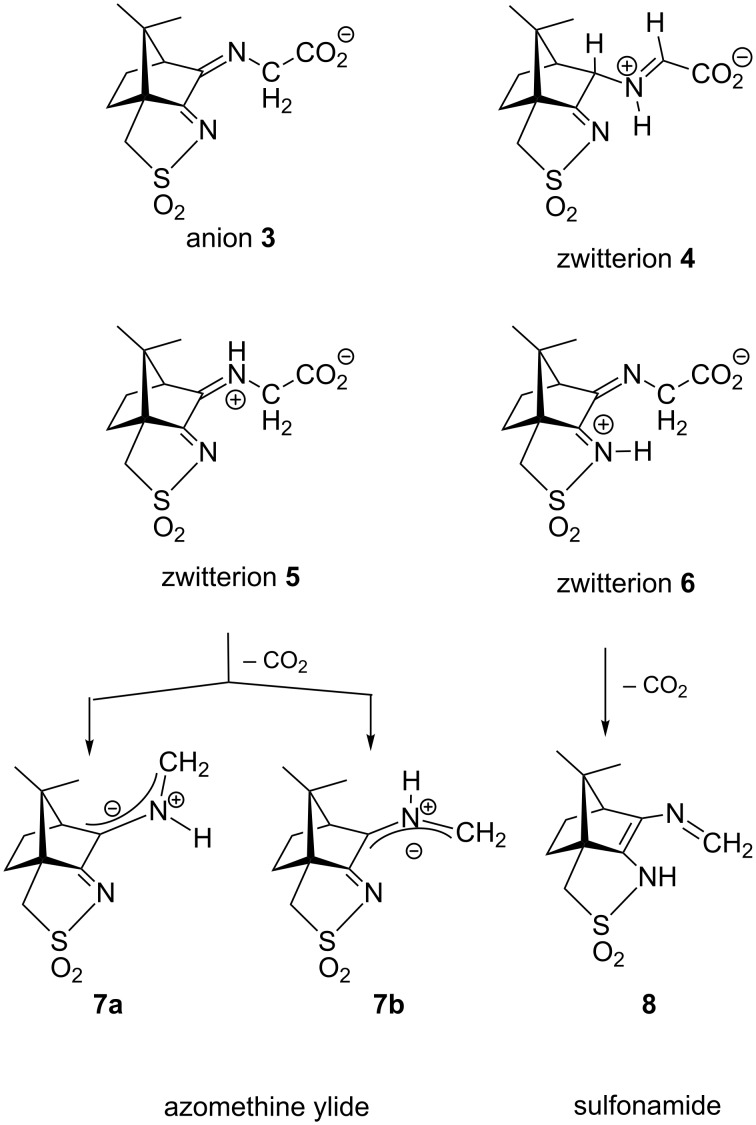
Upper row: anion **3** and zwitterion **4** which are stable upon geometry optimization. Middle row: zwitterions **5** and **6** which lose CO_2_ upon geometry optimization. Lower row: products formed by decarboxylation of the zwitterions.

For imines obtained from reaction of amino acids with ninhydrin, a mechanism of the Strecker degradation was proposed, which includes the anion formed by deprotonation of the carboxyl group as the key intermediate [28–29]. Indeed during geometry optimization we found that the decarboxylation of the anion formed from glycine and ninhydrin occurs without significant energy barrier. This means that the stabilization by delocalization is sufficiently high (Supporting Information File 3), and the suggested mechanism is indeed viable. Geometry optimization of the anion **3** (Figure 7) formed from glycine and oxoimine **1**, did not show any sign of instability. Simple deprotonation is not sufficient to induce the decarboxylation in this case. This difference is probably due to the presence of two neighboring carbonyl groups in ninhydrin which allow for better charge delocalization than does the single sulfonimine group in **1**.

No sign of decarboxylation is observed as well when the conjugation is interrupted as in the zwitterion **4**. The latter would represent the intermediate if a hydride shift occurred in the imine before the decarboxylation step (Figure 7, upper row). In contrast, the zwitterions **5** and **6** readily lose CO_2_ upon geometry optimization, since the negative charge is efficiently compensated in the products **7a**, **7b** and **8**, respectively (Figure 7). A mechanism of the type **5** → **7** (azomethine ylide route) has been proposed for the ninhydrin reaction [30] and other Strecker-type degradation processes [31]. Oxazolidin-5-ones were shown by IR spectroscopy to be formed from amino acids and special carbonyl compounds by cyclization of the imines, and were suggested as intermediates in the formation of azomethine ylides [32]. Analyzing the IR spectra of the reaction mixtures in our reactions did not give any evidence for the occurrence of oxazolidin-5-ones, and calculations on the CO_2_ loss by cycloreversion for the model compound obtained from glycine and glyoxal (see Supporting Information File 3) showed that this reaction has a moderate activation energy but is endothermic and therefore not probable to occur in our reaction conditions.

**Figure 8:**
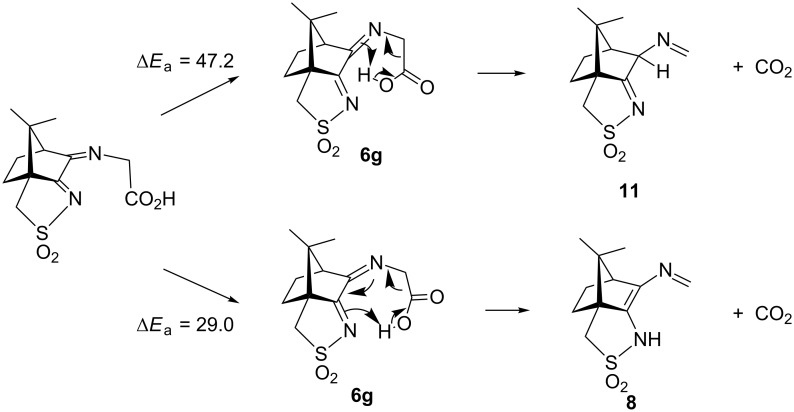
Intramolecular reactions of non-zwitterionic ground state **6g** to **11** (top) or **8** (bottom). The activation energy Δ*E*_a_ denotes the calculated energy difference (in kcal/mol) between the TS and the optimized ground state **6g**. A similar activation barrier was found for the conversion **6g** → **10** (50.3 kcal/mol).

**Figure 9:**
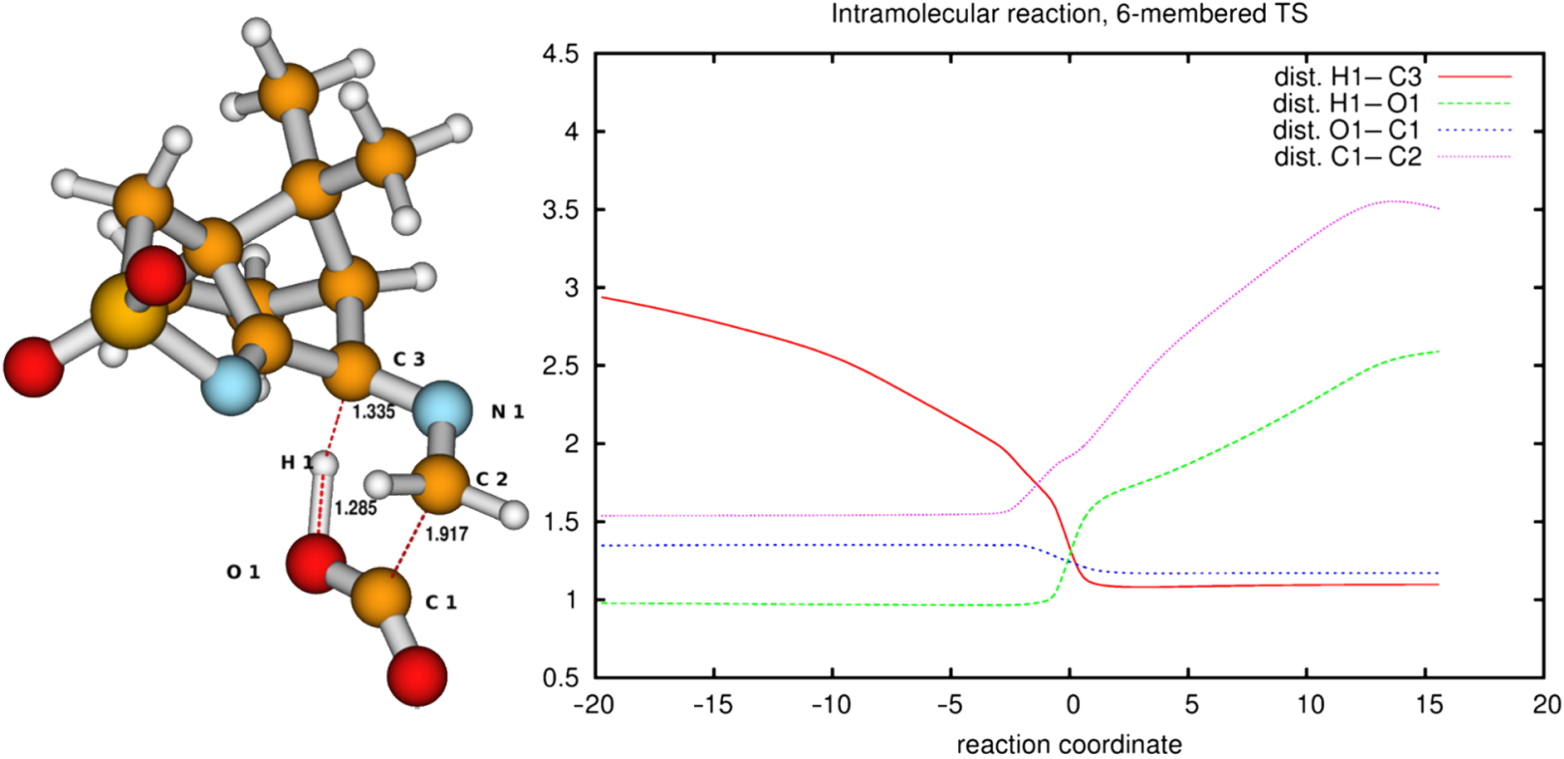
Transition-state geometry and salient bond distances along the IRC path for the reaction of **6g** → **11**. The mass-weighted intrinsic reaction coordinate is in the unit amu^½^*bohr.

Since the barrier towards the Strecker degradation of the zwitterionic forms **5** and **6** is rather due to the formation of these intermediates and not to the degradation itself, we decided to combine both steps in an intramolecular process where a direct transfer of the proton of the carboxyl group to the atom of choice is combined with CO_2_ elimination. Such concerted mechanisms should have an appreciable barrier (activation energy) and therefore give an indication for the viability of different reaction paths. For the ninhydrin reaction with amino acids, a concerted mechanism with simultaneous elimination of CO_2_ and H_2_O from an intermediate aminal (carbinolamine) has been suggested [33], but this could not be confirmed by our calculations (Supporting Information File 3). We next looked at the proposed mechanism for the ninhydrin reaction via a six-membered transition state (TS) which would, when applied to oxoimine **1** and glycine, directly lead to isomers **10** or **11** (Figure 8) as the products [34–35]. We could indeed identify transition states for these reactions. Figure 8 (top) shows the reaction sequence and Figure 9 shows the TS and the change in salient atom distances along the path following the intrinsic reaction coordinate (IRC). A movie is available as Supporting Information File 4.

**Figure 10:**
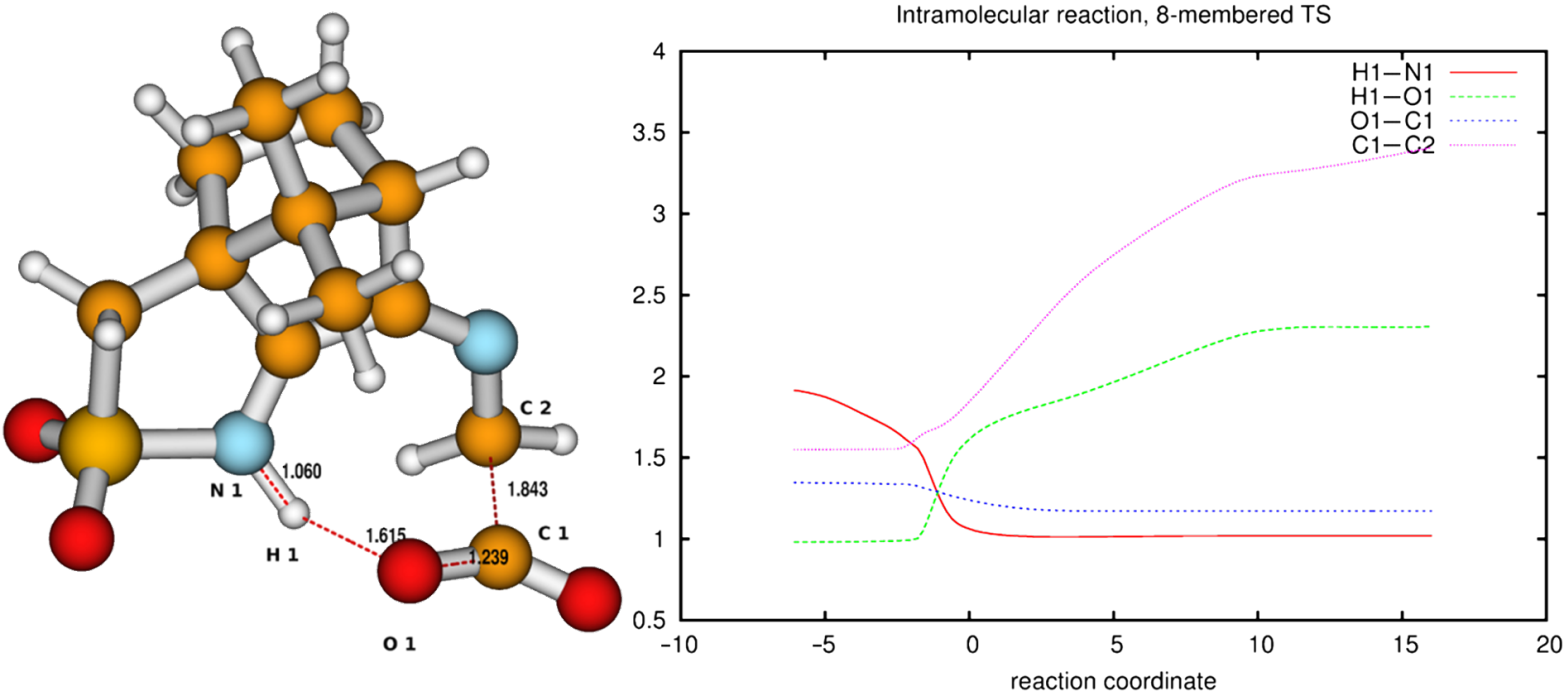
Transition-state geometry and salient bond distances along the IRC path for the reaction of **6g** → **8**. The mass-weighted intrinsic reaction coordinate is in the unit amu^½^*bohr.

The activation barriers for these reactions are very high (≈50 kcal/mol), a fact which clearly speaks against this path. We think that steric effects in general are responsible for the high barriers since the carboxyl group has to approach the bicyclic system from above or below for proton transfer. The situation is different in the second mechanism via an eight-membered ring shown in Figure 8 (bottom). There is no need for the carboxylic group to approach the camphor moiety so closely, and the transfer occurs rather in the periphery of the molecule. Consequently, the activation barrier is considerably lower (∆*E*_a_ = 29.0 kcal/mol). Transition-state geometry and distances along the IRC path are shown in Figure 10, and the corresponding illustrating movie is available Supporting Information File 5.

The transition state leading to compound **8** is considerably closer to the non-ionic ground-state geometry **6g** than its counterpart, as can be seen by comparing the distances of the atoms participating in the degradation reaction (Figure 9 and Figure 10 (right)). This may also contribute to the lower activation barrier in the reaction through the eight-membered TS. In both reactions, bond formation of the hydrogen atom to be transferred to the acceptor atom is done before reaching the TS, while bond breaking occurs at or slightly after in the six-membered TS but before or at the TS in the eight-membered ring. The shortening of the C1–O1 bond to form the double bond of CO_2_ occurs before the TS in both cases.

**Figure 11:**
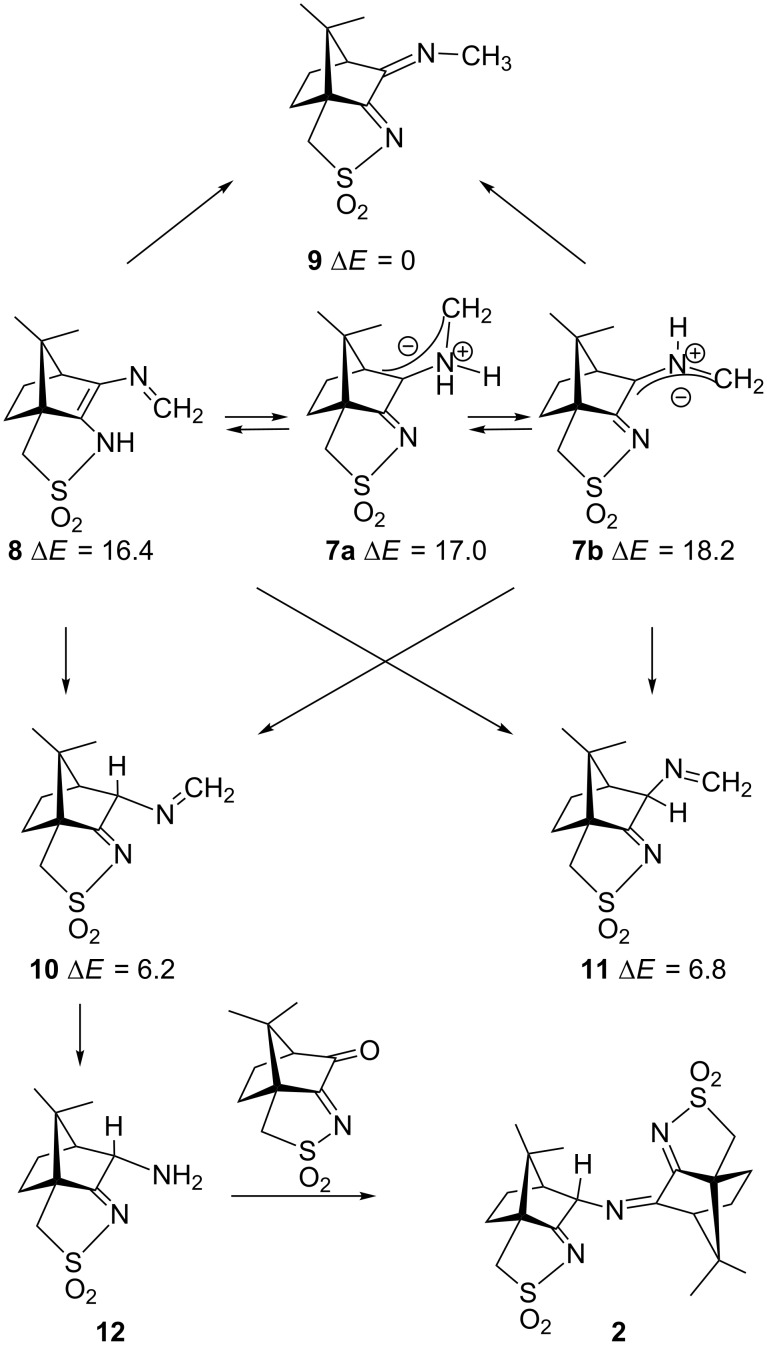
Potential products **7**–**11** of the Strecker degradation together with the reaction of compound **10** to give the compound **2**. ∆*E* denotes the calculated energy difference (in kcal/mol) of the optimized geometries to the most stable one, **9**.

In contrast to these concerted reaction mechanisms, the decarboxylation of the zwitterions **5** and **6** appears to have a very low or near to zero activation barrier and is likely to dominate the reaction pattern. We therefore looked more closely to the products of the decarboxylation reaction (azomethine ylides **7** and ene-sulfonamide **8**). Figure 11 shows the structures and relative energies of geometry-optimized isomeric primary products **7** and **8**, as well as their tautomeric products **9**, **10** and **11** containing the newly formed C–H bonds. These secondary isomers are considerably more stable (10–16 kcal/mol) than the primary products, and once formed, they will probably not tautomerize back to **7** and **8**. Interestingly, the primary products are of quite similar stability (16.4 to 18.2 kcal/mol, respectively), which does not allow a prediction of which path may dominate the decarboxylation. Since the parent zwitterions **5** and **6** cannot be calculated as energy minima, there is no chance to understand, e.g., which conformations of **5** will lead preferentially to **7a** and **7b**, respectively. Since it was found experimentally that the amine **12** is the key intermediate for the formation of compound **2** (Figure 11, bottom), its precursor **10** must be dominant in the expected isomeric mixture formed by tautomerization reactions from the primary products **7** and **8**.

The preferential formation of isomer **10** cannot be explained based on the almost identical thermodynamic energies (6.2 vs 6.8 kcal/mol). We therefore expected that kinetic effects during the tautomerizations might be responsible for the observed selectivity and checked the interconversion pathways between the tautomers **7**–**11** which differ only in the position of one proton. Such a proton migration must of course be catalyzed, and water is an obvious candidate as catalyst. Indeed, water inclusion in reaction paths with high-lying transition states is often observed in water as solvent, as in biochemical reactions, and was demonstrated for, e.g., the analytical detection of sulfenic acids with dimedone [36]. In these systems water can act as proton shuttle. We could identify transition states for water-catalyzed tautomerizations of compounds **7** and **8** to the C–H bonded isomers **9**–**11**. Table 1 shows the calculated activation barriers for these interconversions. Movies of the intrinsic reaction paths for the tautomerizations **7a** → **8** and for **7b** → **10** are shown in Supporting Information File 6 and Supporting Information File 7, respectively.

**Table 1:** Calculated activation energies ∆*E*_a_ (kcal/mol) for the water-catalyzed isomerization of **7**–**11**^a^.

Compound	**7a**	**7b**	**8**	**9**	**10**	**11**

**7a**	–	29.3^b^	8.4	45.4	47.6	43.2
**7b**	28.1^b^	–	–^c^	36.3	33.9	36.1
**8**	10.6	–^c^	–	24.7	35.0	36.2
**9**	48.0	50.5	36.4	–	–	–
**10**	43.3	43.3	42.1	–	–	–
**11**	38.9	45.4	41.6	–	–	–

**^a^**The energies are quoted relative to local energy minima at the potential hypersurface which are reached as endpoints of the IRC calculations. Upper number triangle: reaction from lower to higher compound number; lower number triangle: backward reaction. ^b^Uncatalyzed rotation around C–N bond. ^c^Structure not possible for geometric reasons.

Local minima of the ensemble of a water molecule with compounds **7**–**11** were optimized from the endpoints of the IRC calculations, and the energy differences to the transition states is quoted in Table 1. The calculated activation energies are of course not to be taken literally, since solvent effects were not included, nor was a basis set for superposition error correction applied. We use these calculated values for excluding high-energy pathways, and consider a threshold limit (≈40 kcal/mol) for possible reactions. Thus we can draw the following conclusions from the results:

1. A direct interconversion of **7a** and **7b** (the azomethine ylides) by rotation around the bond which connects the bicyclic ring system with the nitrogen atom appears possible (barrier of medium height). No interference of water is necessary for this step.

2. The fastest tautomerization in the reaction mixture occurs between **7a** and **8** (the ene-sulfonamide). The low barriers (8.4 and 10.6 kcal/mol) should allow the equilibrium to be established rapidly. On the other hand, there is no possibility of proton transfer from **7b** to **8** directly, but only via isomer **7a** which is formed according to 1).

3. Barriers of medium height are observed for the tautomerizations **7b** → **9** + **10** + **11** and **8** → **9** + **10** + **11**. No direct reaction to these products is possible from **7a**, but only after interconversion to **7b** according to 1. From **8**, there should be a preference for the methyl compound **9** as major product which hydrolyzes back to the oxoimine **1**. This could be seen as an oxoimine-catalyzed decomposition of the amino acid to methylamine and CO_2_. Even after complete consumption of the starting oxoimine by imine formation with the amino acid, this reaction could be a source of **1** which is required for the formation of compound **2** (Figure 11, bottom).

Compound **2** is obtained from **1** and the amine **12** which in turn is formed by hydrolysis of the imine **10**. No product analogous to **2** which could be derived from the isomeric amine formed by hydrolysis of **11** was detected. This observation suggests that **10** is formed preferentially, and thus the key intermediate is rather **7b** and not **8**. Such proposal is supported by the calculated activation barrier for the formation of **10** from **7b** which is slightly lower (2.2 kcal/mol) than the corresponding barrier to form **11** and **9** from **7b**. To confirm this, higher level calculations or further experimental evidence would be required.

#### Experimental support by ESIMS

**Figure 12:**
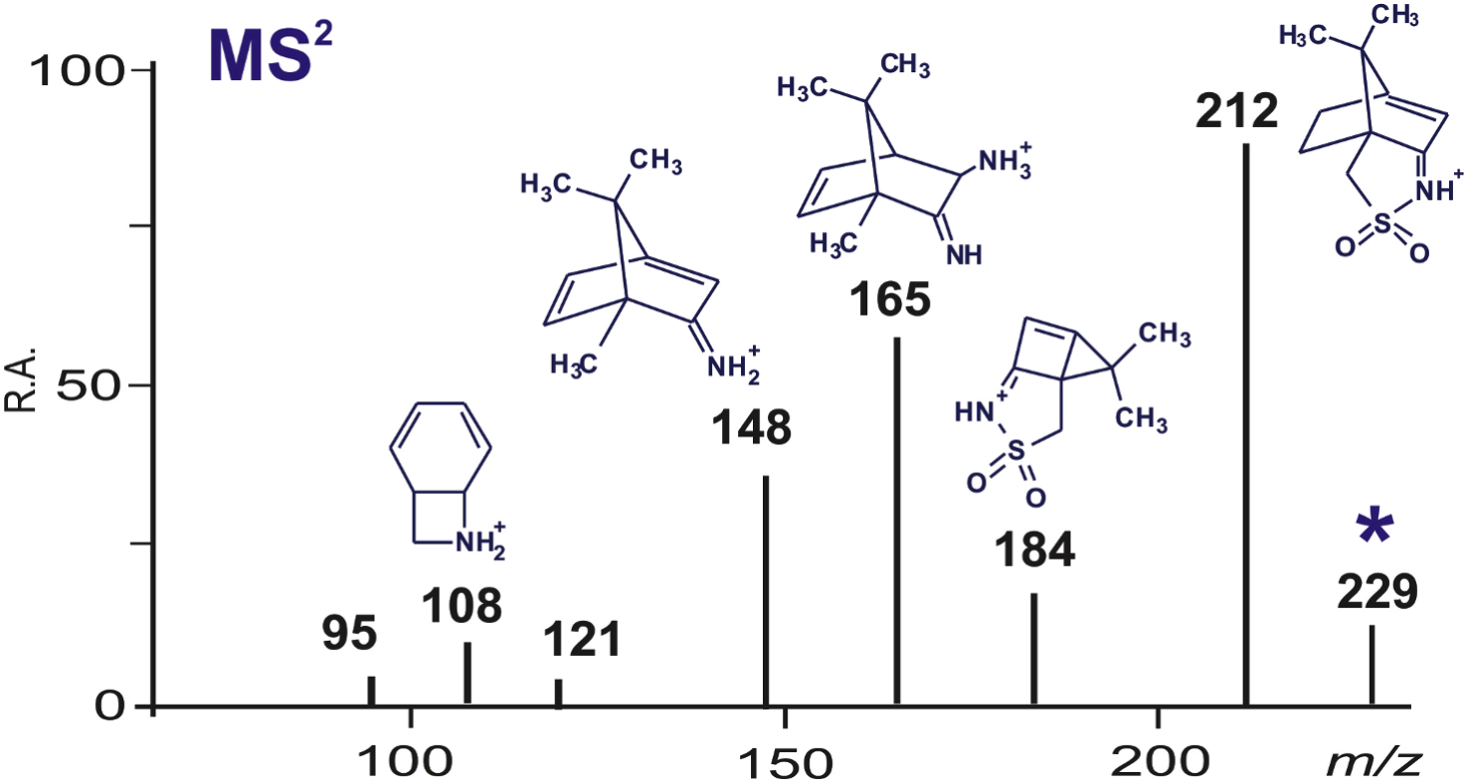
ESI(+) tandem mass spectrum of the intermediate **12** (*m*/*z* 229) and proposed fragment ions.

Looking for an experimental support to validate the intermediates proposed in Figure 11, the reaction of **1** with L-phenylalanine was monitored by ESIMS at different reaction times. In all reaction mixtures the positive ESI(+) mass spectra showed a peak at *m*/*z* 229 which was further studied by collision-induced dissociations (CID). The main fragments observed in the MS^2^ spectrum (Figure 12) support the formation of an amine (**12** or its isomer with (*R*)-3A configuration). See Supporting Information File 1, Figures S9 and S10 for the full spectrum and proposed mechanism, respectively.

## Conclusion

From the reaction of amino acids with the camphor-derived oxoimine **1**, compound **2** was isolated and spectroscopically characterized as being formed by two camphor moieties connected by a =N– group. This group is the only part of **2** which originates from the amino acid, a fact which relates the structure with that of Ruhemann's purple, the colored compound formed in the reaction of ninhydrin with amino acids. This means that a Strecker degradation of the amino acid is involved. In order to better understand the reaction mechanism, we investigated the structure of potential products, intermediates and transition states by DFT calculations. The first step is the formation of imines of the amino acids with the C=O group. These are stable as neutral compounds as well as carboxylate anions. This is different in the ninhydrin reaction where the corresponding anions lose CO_2_ upon attempted geometry optimization. Decarboxylation of the imines of the oxoimine **1** only occurs when a zwitterion is formed by proton transfer to one of the two nitrogen atoms. This lower reactivity when compared to ninhydrin is due to the reduced ability of the remaining molecule to stabilize the negative charge which is transferred to it after the loss of CO_2_ from the carboxylate group. This charge can be delocalized only over two double bonds and not over three plus the aromatic system as in ninhydrin. The resulting intermediates, azomethine ylides or ene-sulfonamide, undergo water-catalyzed tautomerization reactions followed by hydrolysis of the C=N bonds to form amines with a new chiral center at the former C=O group of compound **1**. The isolated compound **2** is derived from one of these isomeric amines.

## Experimental

The amino acids, (1*S*)-(+)-10-camphorsulfonic acid and the solvents (PA grade) were purchased from Sigma-Aldrich and used without further purification. EtOH was purchased from Panreac. Oxoimine **1** ((3a*S*)-8,8-dimethyl-5,6-dihydro-3*H*-3a,6-methano-2,1-benzoisothiazol-7(4*H*)-one 2,2-dioxide) was prepared from (1*S*)-(+)-10-camphorsulfonic acid following the published procedure [3]. The IR spectra were obtained from KBr pellets using a JASCO FT/IR 4100 spectrometer. NMR spectra (^1^H, ^13^C, DEPT, HSQC, HMBC, NOESY) were obtained from CD_3_CN solutions using a Bruker Avance II^+^ 400 MHz spectrometer. The chemical shifts were referenced to TMS (δ = 0 ppm). LRESI mass spectrometry and tandem mass experiments were carried out on a LCQ Fleet mass spectrometer operating in the ESI positive ion mode (Thermo Scientific). The optimized parameters were as follows: ion spray voltage, +4.5 kV; capillary voltage, 16 V; tube lens offset, −63 V; sheath gas (N_2_), 80 arbitrary units; auxiliary gas, 5 arbitrary units; capillary temperature, 250 °C. The spectra were recorded in the range 100–1500 Da and a spectrum typically corresponds to the average of 20–35 scans. Tandem mass spectra were obtained with an isolation window of 2 Da, a 30% relative collision energy and with an activation time of 30 ms. The HRESI mass spectrum was obtained on a UHR-QqTOF Impact II (Bruker Daltonics) operating in the high resolution mass mode.

### Synthesis

Reaction of amino acids with **1**: Acetic acid (200 μL) was added to the suspension of oxoimine **1** (0.23 g, 1.01 mM) in EtOH (5 mL) and the mixture was stirred at 45 °C for 0.25 h. Then, the α-amino acid (glycine; L-alanine; L-phenylalanine; leucine) in ca. 2.5 stoichiometric excess was dissolved in H_2_O (ca. 4 mL, 60 °C) and added. The suspension was stirred at 45 °C for ca. 10 h. Compound **2** ((3a*S*,6*S*,*Z*)-7-(((3a*S*,6*S*)-8,8-dimethyl-2,2-dioxido-4,5,6,7-tetrahydro-3*H*-3a,6-methanobenzo[*c*]isothiazol-7-yl)imino)-8,8-dimethyl-4,5,6,7-tetrahydro-3*H*-3a,6-methanobenzo[*c*]isothiazole 2,2-dioxide) was obtained as a pale yellow solid upon filtration and drying. Yield of **2**: 40%. Anal. calcd for C_20_H_27_N_3_O_4_S_2_·0.5EtOH: C, 54.7; N, 9.1; H, 6.5; S, 13.9; found: C, 54.8; N, 9.2; H, 6.3; S, 14.1; IR (cm^−1^): 1676, 1641 (ν_CN_), 1338 (ν_SO2asym_), 1208 (ν_C-N_), 1162 (ν_SO2sym_); ^1^H NMR (253 K, 400 MHz, CD_3_CN) δ 4.81 (*d*, ^3^*J*_3A4A_ = 4.37 Hz, 1H, H3A), 3.51 (*d*, ^2^*J*_8Bexo8Bendo_ = 13.8 Hz, 1H, H8B1), 3.42 (*d*, ^2^*J*_8Aexo8Aendo_ = 13.8 Hz, 1H, H8A1), 3.28 (*m*, 1H, 4B), 3.27 *d*, ^2^*J*_8Bexo8Bendo_ = 13.8 Hz, 1H, H8B2), 3.13 (*d*, ^2^*J*_8Aexo8Aendo_ = 13.8 Hz, 1H, H8A2), 2.43 (*t*, ^3^*J*_3A4A_ = 4.7 Hz, 1H, H4A), 2.22 (*m*, 3H, H6A2+H5B1+H6B2), 2.07 (*m*, 1H, H5A1), 1.84 (*m*, 1H, H5A2), 1.78 (*m*, 2H, H5B2+H6B1), 1.61 (*m*, 1H, H6A1), 1.11 (s, 3H, H9A), 1.07 (s, 3H, H9B), 0.95 (s, 3H, H10A), 0.83 (s, 3H, H10B); ^13^C{^1^H} NMR (253 K, 100 MHz, CD_3_CN) δ 194.9 (C2A), 186.4 (C2B), 173.3 (C3B), 65.3 (C1A), 64.4 (C3A), 63.9 (C1B), 51.2 (C4B), 51.6 (C4A), 50.7 (C8A), 49.9 (C8B), 47.3 (C7A), 46.7 (C7B), 29.5 (C6A), 28.2 (C6B), 24.5 (C5B), 19.5 (C10B), 19.4 (C5A), 18.9 (C9A), 18.5 (C10A), 17.8 (C9B); HRMS–ESI (*m*/*z*): [M + H]^+^ calcd for C_20_H_28_N_3_O_4_S_2_, 438.1516; found, 438.1506.

### Calculations

Geometry optimizations, Hessian calculations, saddle point searches and intrinsic reaction path (IRC) calculations [37–39] were done with the program PCGamess (version 7.1) [40–41], using B3LYP/6-31G** for neutral compounds and B3LYP/6-31++G** for anions. Zero-point energies and basis set superposition errors were not included since no high precision numerical results were needed. Data analysis and visualization was done with Molden [42].

## Supporting Information

File 1 Experimental spectra for compound **2**: FTIR, NMR (^1^H, ^13^C, DEPT, HMBC, HSQC, NOESY), ESIMS and the proposed fragmentation mechanism for **2** and **12**.

File 2 Calculations of the NMR chemical shifts for the assignment of the conﬁguration at carbon atom 3A in compound **2**.

File 3 Calculations for Strecker degradation via zwitterions in the case of glyoxal and ninhydrin.

File 4 Calculated reaction (IRC path) via the transition state **6g** → **11**.

File 5 Calculated reaction (IRC path) via the transition state **6g** → **8**.

File 6 Calculated reaction (IRC path) via the transition state **7a** → **8**.

File 7 Calculated reaction (IRC path) via the transition state **7b** → **10**.

File 8 Calculated reaction (IRC path) via the transition state glyoxal/glycine, CO_2_ loss.

File 9 Calculated reaction (IRC path) via the transition state imine ninhydrine/glycine, zwitterion, azomethine ylide formation.

File 10 Calculated reaction (IRC path) via the transition state imine ninhydrine/glycine, CO_2_ loss.
