# Supplementary material for: Strecker degradation of amino acids promoted by a camphor-derived sulfonamide
Source: Beilstein J Org Chem. 2016 Apr 18;12:732–44. doi: 10.3762/bjoc.12.73 (PMC4902088; doi:10.3762/bjoc.12.73)
Supplement: File 1 — Experimental spectra for compound 2: FTIR, NMR (1H, 13C, DEPT, HMBC, HSQC, NOESY), ESIMS and the proposed fragmentation mechanism for 2 and 12. [file Beilstein_J_Org_Chem-12-732-s001.pdf]

Supporting Information File 1

for

Strecker degradation of amino acids promoted by a camphor-derived sulfonamide

M. Fernanda N. N. Carvalho[1]*, M. João Ferreira[1], Ana S. O. Knittel[1], Maria da Conceição Oliveira[1], João Costa Pessoa[1], Rudolf Herrmann[2], and Gabriele Wagner[3]

Address: [1]Centro de Química Estrutural, Instituto Superior Técnico, Universidade de Lisboa, Av. Rovisco Pais 1049-001 Lisboa, Portugal, [2]Institut für Physik, Universität Augsburg, Universitätsstr. 2, 86159 Augsburg, Germany and [3]University of Chester, Faculty of Science and Engineering, Department of Natural Sciences, Thornton Science Park, Pool Lane, Ince, Chester, CH2 4NU, UK

Email: Maria Fernanda N. N. Carvalho - fcarvalho@ist.utl.pt

*Corresponding author

Experimental spectra for compound **2**: FTIR, NMR (^{1}H, ^{13}C, DEPT, HMBC, HSQC, NOESY), ESIMS and the proposed fragmentation mechanism for **2** and **12**

Experimental Data

(3a*S*,6*S*,*Z*)-7-(((3a*S*,6*S*)-8,8-Dimethyl-2,2-dioxido-4,5,6,7-tetrahydro-3*H*-3a,6-methanobenzo[*c*]isothiazol-7-yl)imino)-8,8-dimethyl-4,5,6,7-tetrahydro-3H-3a,6-methanobenzo[*c*]isothiazole 2,2-dioxide (**2**).

Spectroscopic data

FTIR

The spectrum was obtained from KBr pellets using a JASCO FT/IR 4100 spectrometer.

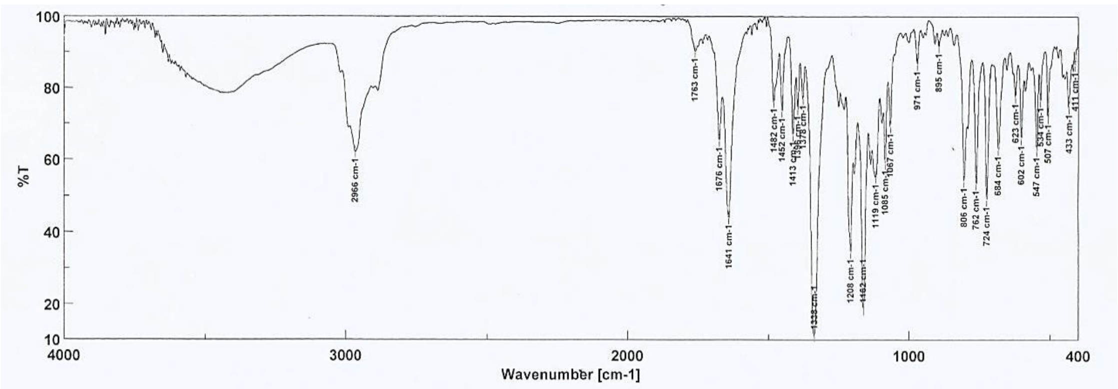

Figure S1: Spectrum of **2** (range 4000–400 cm^{-1})

NMR

The spectra were obtained at 20 °C in CD_3CN using a Bruker Avance II^{+} 400 MHz spectrometer. The chemical shifts were referenced to TMS (δ = 0 ppm).

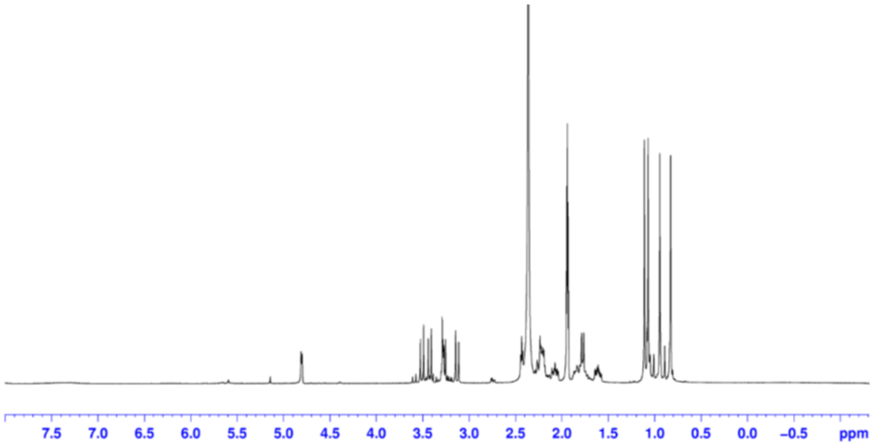

Figure S2: ^{1}H NMR spectrum of **2** obtained in CD_3CN at−20 °C.

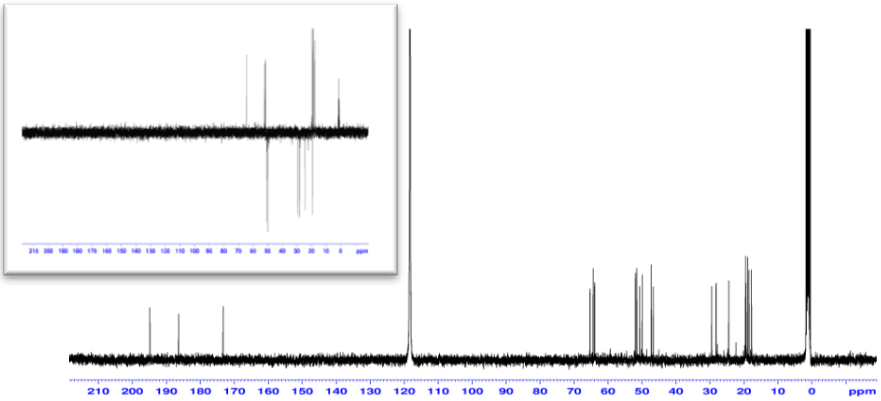

Figure S3: ^{13}C NMR spectrum and DEPT (insert) of **2** obtained in CD_3CN at −20 °C.

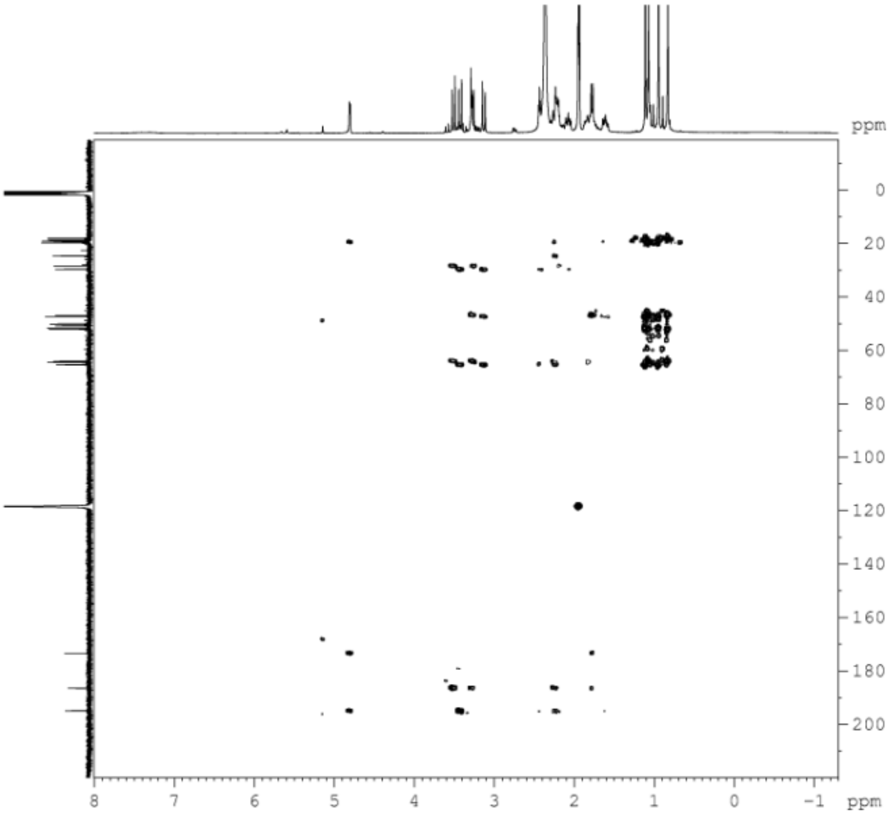

Figure S4: HMBC of **2** obtained in CD_3CN at −20 °C.

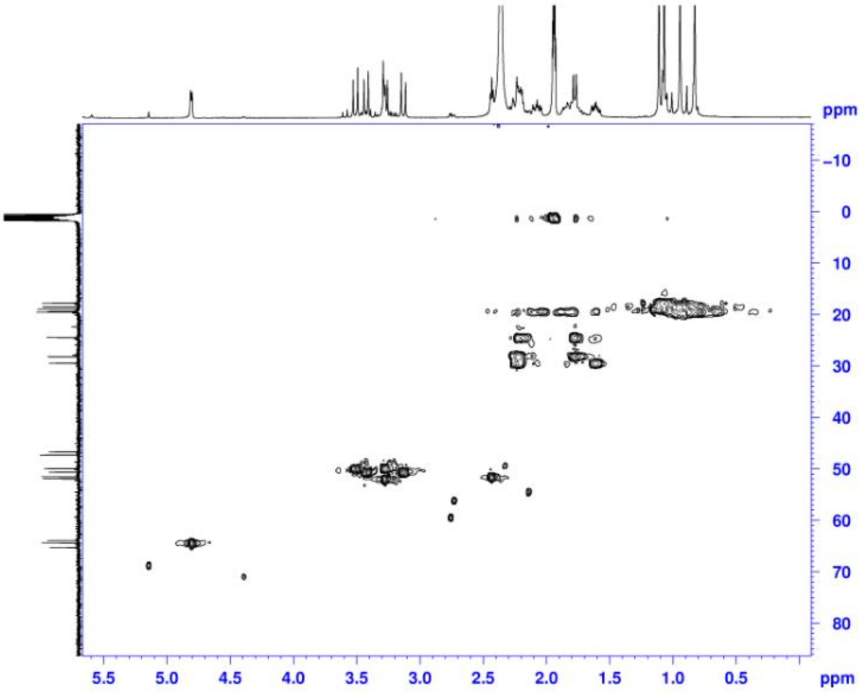

Figure S5: HSQC of **2** obtained in CD_3CN at −20 °C.

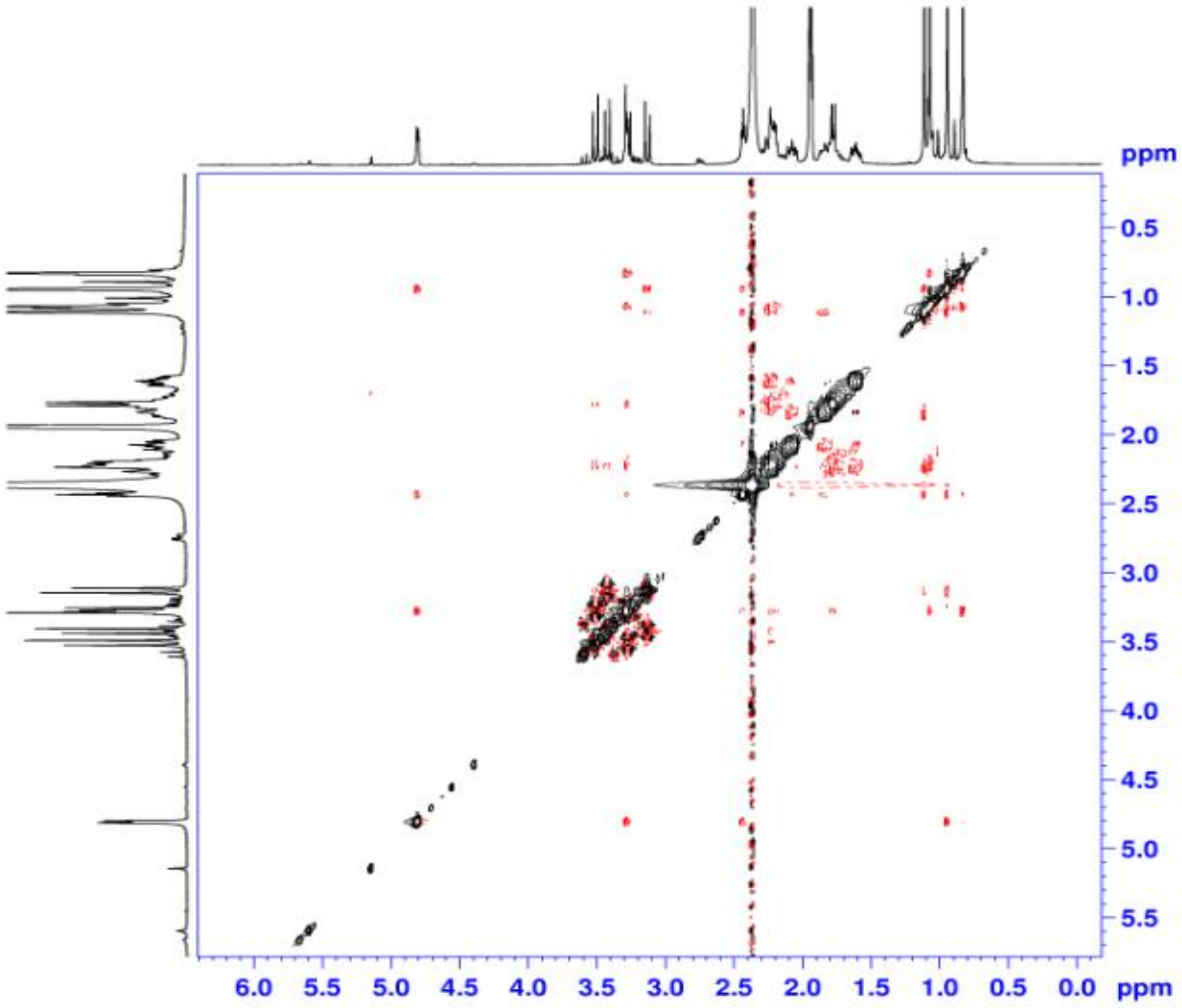

Figure S6: NOESY of **2** obtained in CD_3CN at −20 °C.

ESI-MS

Data for **2**

LRESI mass spectrum and tandem mass experiments were carried out on a LCQ Fleet mass spectrometer operated in the ESI positive ion mode (Thermo Scientific), with the following optimized parameters: ion spray voltage, +4.5 kV; capillary voltage, 16 V; tube lens offset, −63 V; sheath gas (N_2), 80 arbitrary units; auxiliary gas, 5 arbitrary units; capillary temperature, 250 °C. The spectra were recorded in the range 100–1500 Da. Spectrum typically corresponds to the average of 20–35 scans. Tandem mass spectrum was obtained with an isolation window of 2 Da, a 30% relative collision energy and with an activation energy of 30 ms. HRESI mass spectrum was obtained on a UHR-QqTOF Impact II (Bruker Daltonics) operating in the high resolution mode.

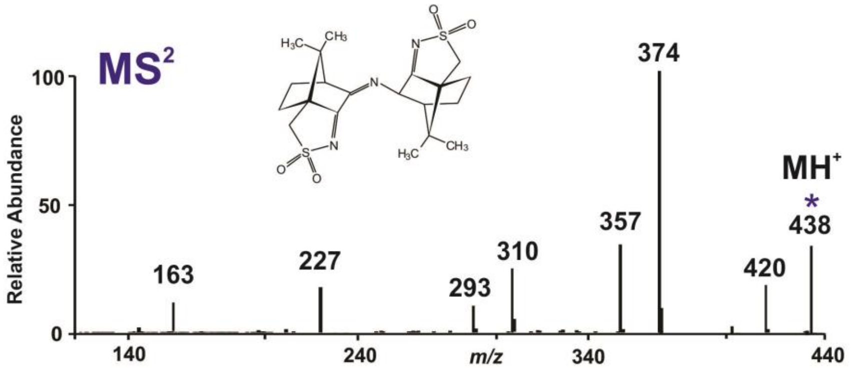

Figure S7: ESI tandem mass spectrum of the protonated molecule of **2.**

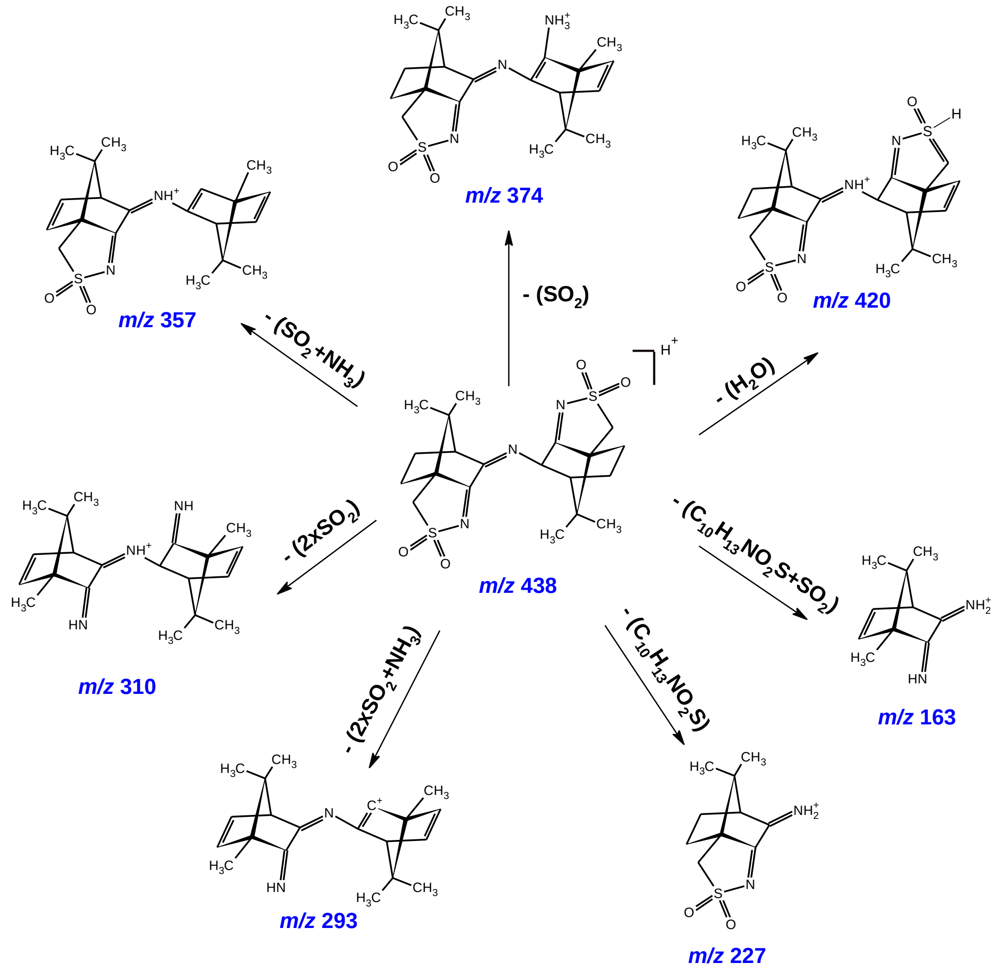

Figure S8: Proposed fragmentation mechanism for the precursor ion *m/z* 438.

Data for **12**

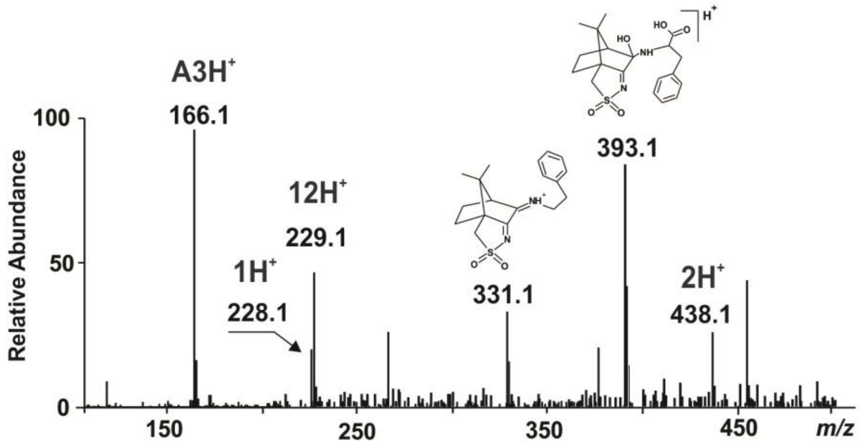

Figure S9: ESI tandem mass spectrum of the reaction mixture after 1 h reaction showing protonated phenylalanine, **A3**; oxoimine, **1**; the proposed intermediate **12** (Figure 11 in the manuscript) and the final product **2**. Other intermediates were identified and their formula assigned to the corresponding mass.

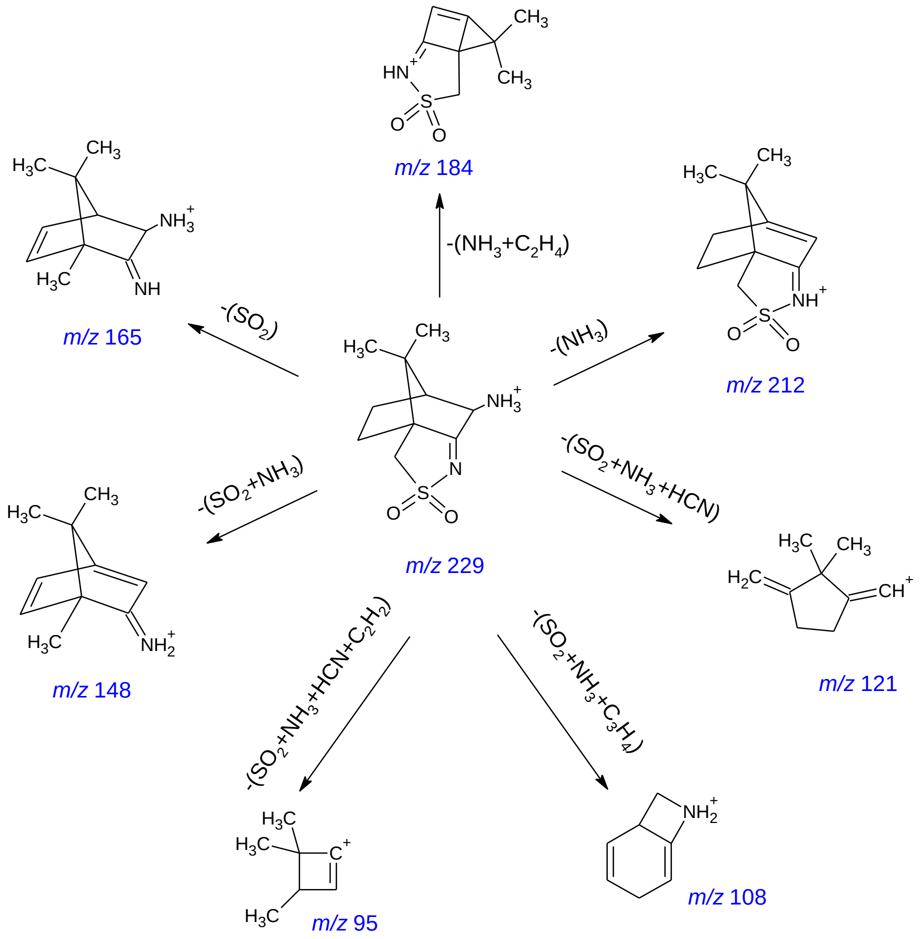

Figure S10: Proposed fragmentation mechanism for intermediate **12** (*m/z* 229).
