# Supplementary figures and images for: Strecker degradation of amino acids promoted by a camphor-derived sulfonamide
Source: Beilstein J Org Chem. 2016 Apr 18;12:732–44. doi: 10.3762/bjoc.12.73 (PMC4902088; doi:10.3762/bjoc.12.73)

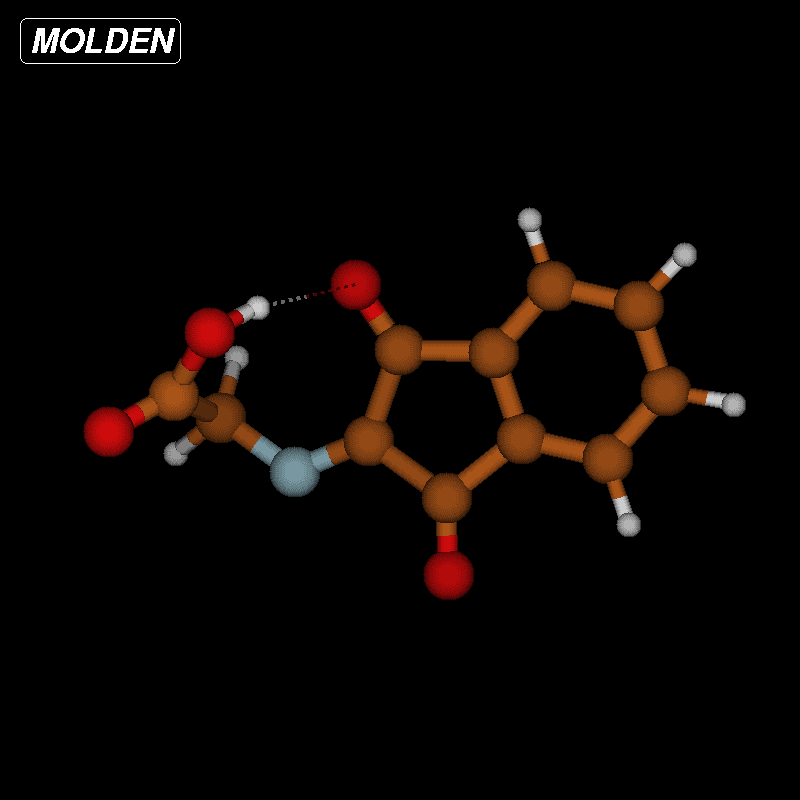

Supplement: File 2 — Calculations of the NMR chemical shifts for the assignment of the conﬁguration at carbon atom 3A in compound 2. [file Beilstein_J_Org_Chem-12-732-s002.gif]

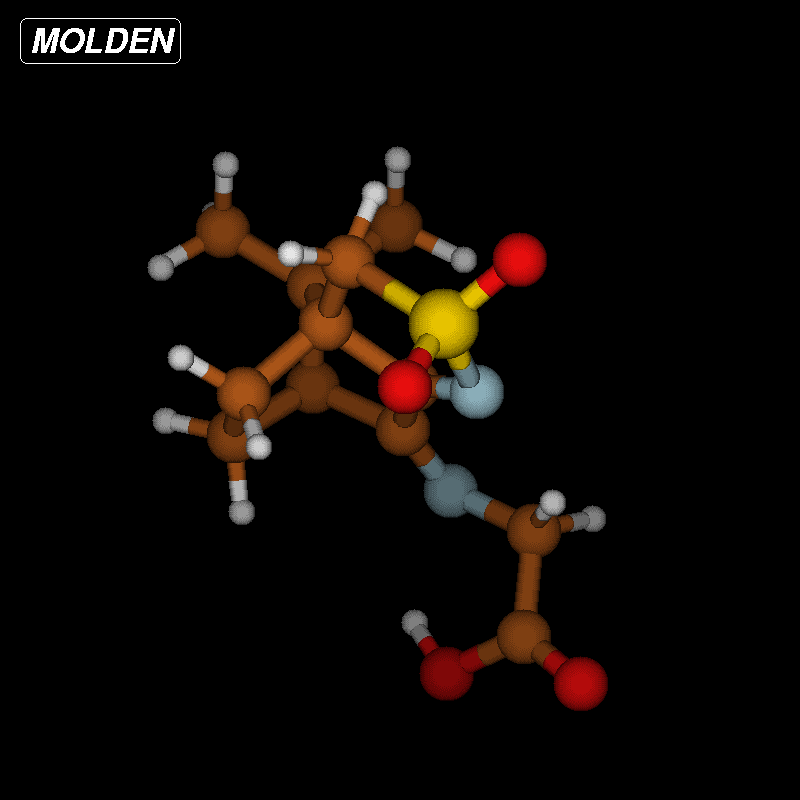

Supplement: File 5 — Calculated reaction (IRC path) via the transition state 6g → 8. [file Beilstein_J_Org_Chem-12-732-s005.gif]

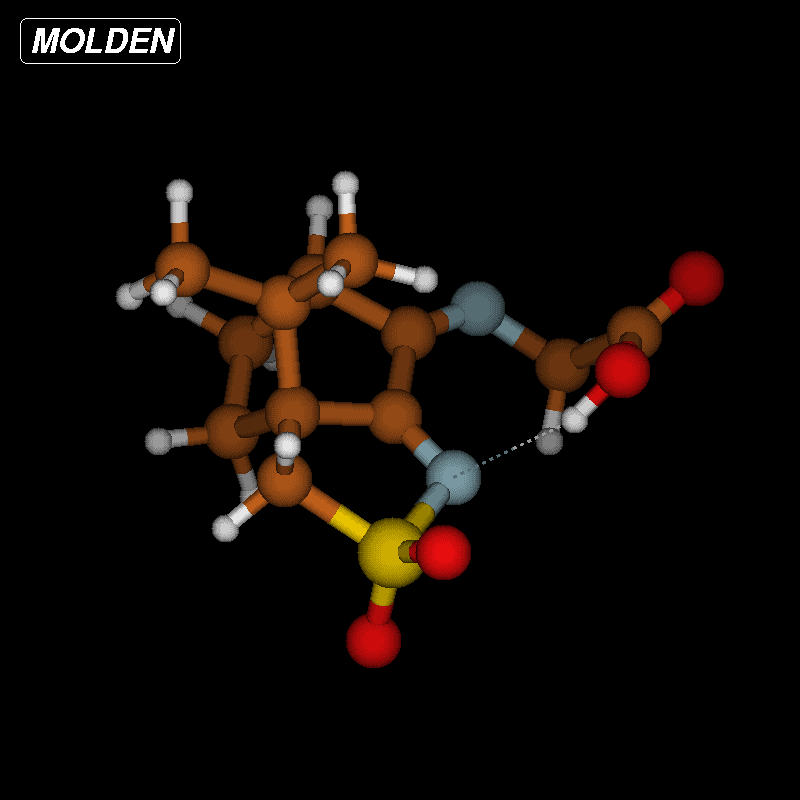

Supplement: File 6 — Calculated reaction (IRC path) via the transition state 7a → 8. [file Beilstein_J_Org_Chem-12-732-s006.gif]

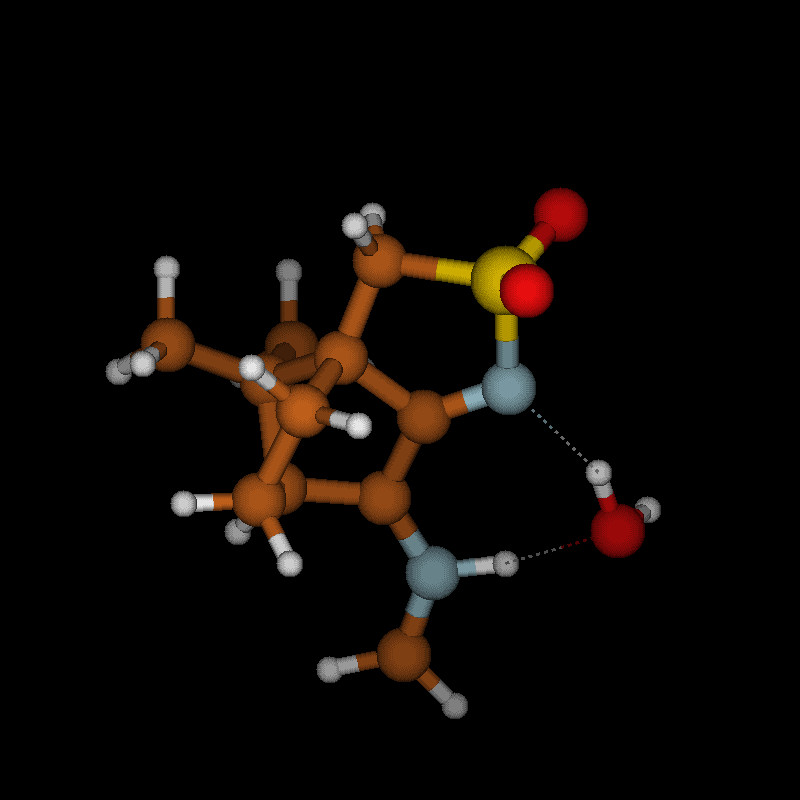

Supplement: File 7 — Calculated reaction (IRC path) via the transition state 7b → 10. [file Beilstein_J_Org_Chem-12-732-s007.gif]

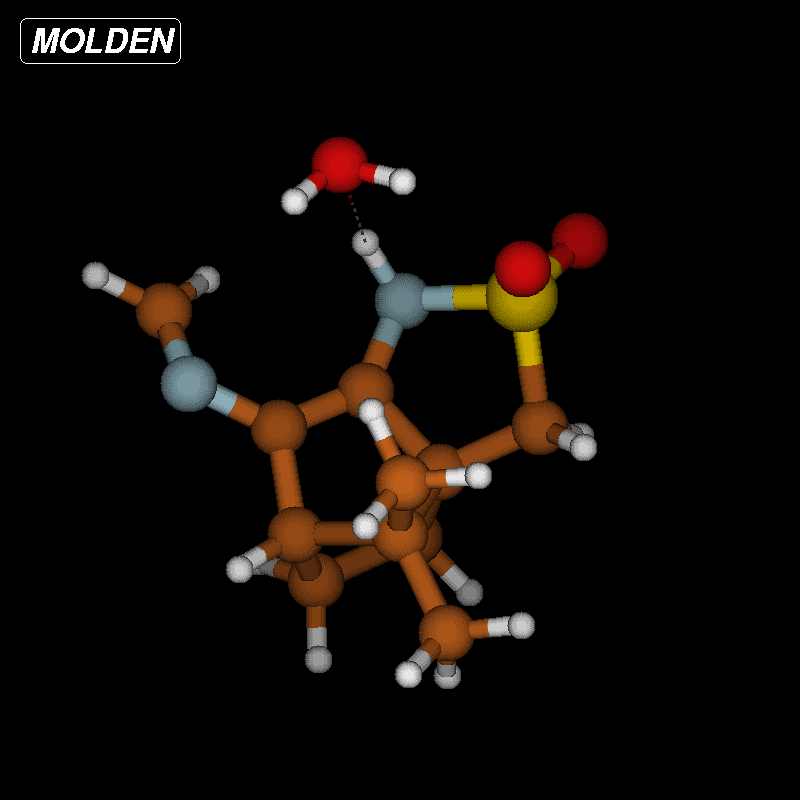

Supplement: File 8 — Calculated reaction (IRC path) via the transition state glyoxal/glycine, CO2 loss. [file Beilstein_J_Org_Chem-12-732-s008.gif]

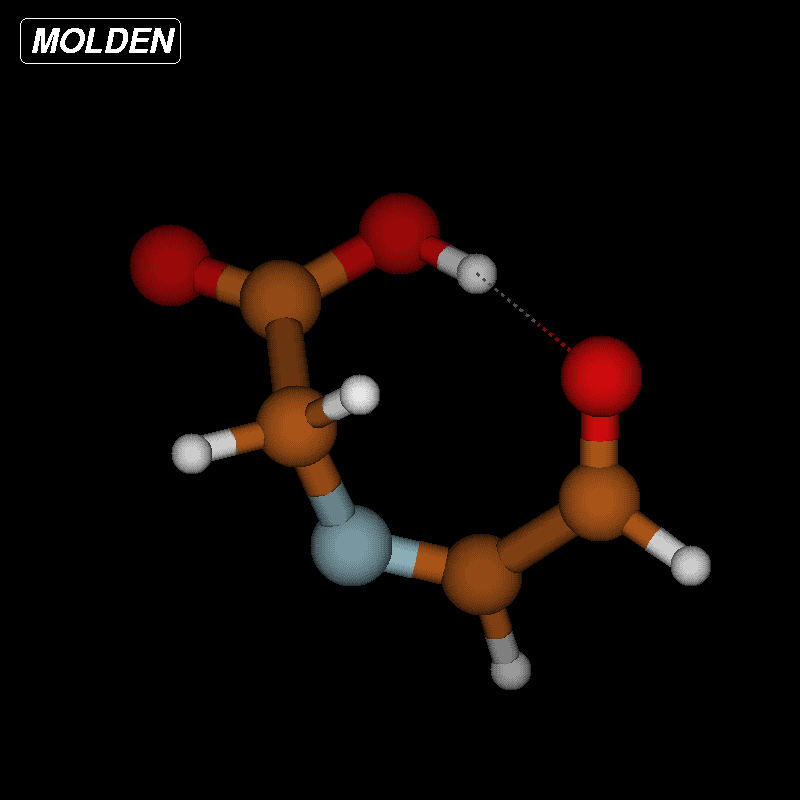

Supplement: File 9 — Calculated reaction (IRC path) via the transition state imine ninhydrine/glycine, zwitterion, azomethine ylide formation. [file Beilstein_J_Org_Chem-12-732-s009.gif]

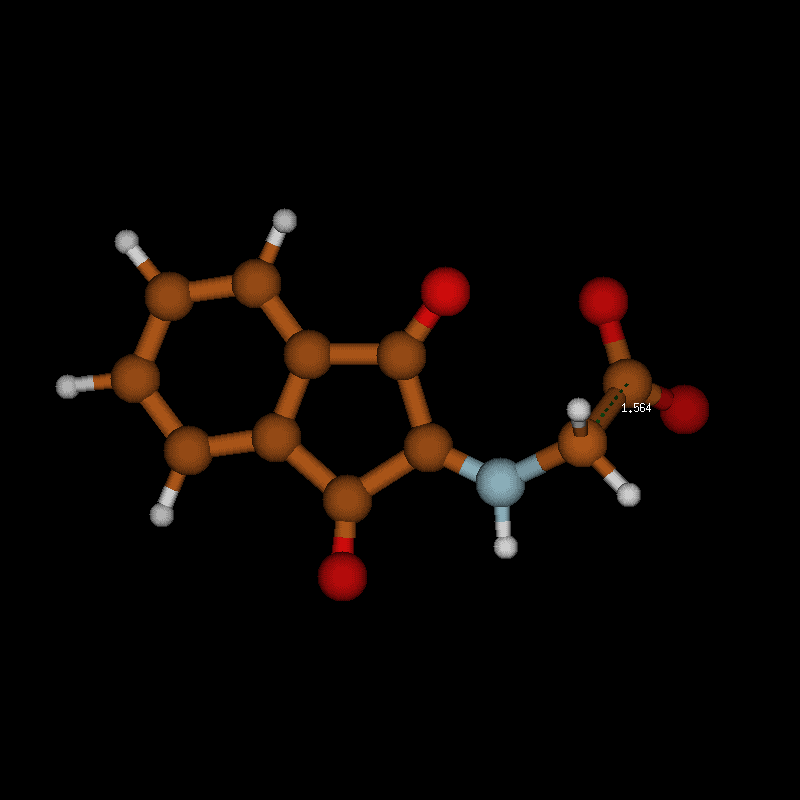

Supplement: File 10 — Calculated reaction (IRC path) via the transition state imine ninhydrine/glycine, CO2 loss. [file Beilstein_J_Org_Chem-12-732-s010.gif]
