# Supplementary material for: Strecker degradation of amino acids promoted by a camphor-derived sulfonamide
Source: Beilstein J Org Chem. 2016 Apr 18;12:732–44. doi: 10.3762/bjoc.12.73 (PMC4902088; doi:10.3762/bjoc.12.73)
Supplement: File 3 — Calculations for Strecker degradation via zwitterions in the case of glyoxal and ninhydrin. [file Beilstein_J_Org_Chem-12-732-s003.pdf]

Supporting Information File 2

for

Strecker degradation of amino acids promoted by a camphor-derived sulfonamide

M. Fernanda N. N. Carvalho[1]*, M. João Ferreira[1], Ana S. O. Knittel[1], Maria da Conceição Oliveira[1], João Costa Pessoa[1], Rudolf Herrmann[2], and Gabriele Wagner[3]

Address: [1]Centro de Química Estrutural, Instituto Superior Técnico, Universidade de Lisboa, Av. Rovisco Pais 1049-001 Lisboa, Portugal, [2]Institut für Physik, Universität Augsburg, Universitätsstr. 2, 86159 Augsburg, Germany and [3]University of Chester, Faculty of Science and Engineering, Department of Natural Sciences, Thornton Science Park, Pool Lane, Ince, Chester, CH2 4NU, UK

Email: Maria Fernanda N. N. Carvalho - fcarvalho@ist.utl.pt

*Corresponding author

Calculations of the NMR chemical shifts for the assignment of the configuration at carbon atom 3A in compound **2**

NMR chemical shift calculations for the assignment of configurations in camphor derivatives

Geometry optimizations and Hessian calculations were done with the program PC GAMESS (version 7.1) [1,2], using B3LYP/6-31G**. NMR chemical shifts were calculated with NWChem (version 6.3) [3] using the GIAO method (gauge including atomic orbitals) [4] with B3LYP/6-311++G**, including solvent effects by the Cosmo method (ε = 4.9 for chloroform and 36.6 for acetonitrile) [5]. Data analysis and visualization was done with MOLDEN [6].

1. Establishing a correlation between calculated and experimental NMR chemical shifts for a standard compound, the oxoimine **1** [7].

Structure elucidation of organic compounds by NMR is often a cumbersome task because of overlap of signals and quite small chemical shift differences between isomeric (particularly diastereoisomeric) compounds. The comparison of measured NMR data with calculated values can be very helpful and has already led to revised structure assignments of natural products [8]. In general, experimental chemical shifts are measured in solution, and often in solvents of medium polarity like chloroform at room temperature. In contrast, calculations are normally for isolated molecules ("gas phase") at 0 K. One can therefore not expect to obtain identical numerical values. Instead, linear regression was used to obtain correlations. These may be used for deciding which calculated structure does better fit to the experimental data and supports assignments for diastereoisomers [9,10]. Other approaches use a database of well-studied compounds to establish a correlation which is then applied to other molecules, both for ^{13}C [11] and ^{1}H chemical shifts [12]. Unspecific solvent effects can be accounted for by PCM (polarizable continuum method) [12,13,14]. As to computational techniques, the GIAO (gauge including atomic orbital) method is increasingly preferred [4,11,12]. The computations can be kept at moderate levels; many authors noted that geometry optimization at the B3LYP/6-31G** level is sufficient, and even the GIAO calculation gives reasonable results at this level at least for ^{13}C [8,10,11,15,16] although basis sets with triple-ζ quality have been recommended for distinguishing complex macrocyclic natural products [13] or even for comparatively simple organic molecules [12,14].

Given this situation, we decided to proceed in the following way: We chose not to rely on a database of compounds but to use a simple standard molecule as starting point which has close structural similarity to the compounds to be investigated. An obvious choice was the oxoimine **1** [7] which was also the principal starting material in the syntheses. Its ^{1}H and ^{13}C NMR spectra in $CDCl_3$ and CD_3CN could be fully assigned experimentally and the data are given in Table S1, together with the calculated shift values for the best of the tested computation methods (B3LYP/6-311++G**) which was also recommended by others [17,18]. The combination of B3LYP with other Pople basis sets was less satisfactory. Surprisingly, the recently developed SSB-D functional [19,20] which was recommended for NMR computations, showed a slightly lower R^2 value than B3LYP in the correlations shown below. We decided not to include the carbon atom directly connected to the sulfur atom in the correlation since such atoms are rather problematic in calculations [14] and would deteriorate the validity of the scaling equation to be derived.

Table S1: Calculated and experimental values of the chemical shifts of carbon and hydrogen atoms in the oxoimine **1** in chloroform and acetonitrile. The numbering of the carbon atoms is

shown in Figure S11.

Carbon nr.	chloroform				acetonitrile				H sterochem.
	calc..		exp.		calc.		exp.		
	^{13}C	^{1}H	^{13}C	^{1}H	^{13}C	^{1}H	^{13}C	^{1}H	
1	72.4	—	63.0	—	73.3	—	64.2	—	
2	192.5	—	181.6	—	194.5	—	183.9	—	
3	216.6	—	198.1	—	218.5	—	200.0	—	
4	65.8	2.74	59.3	2.76	66.8	2.84	60.1	2.76	
5	28.5	2.40<br>1.78	22.4	2.28<br>1.82	28.8	2.49<br>1.81	22.9	2.30<br>1.86	exo<br>endo
6	35.1	2.34<br>2.74	28.2	2.26<br>1.98	35.5	2.45<br>1.90	28.5	2.30<br>1.94	exo<br>endo
7	54.6	—	44.7	—	55.0	—	45.5	—	
8	70.0	3.19<br>3.45	50.2	3.22<br>3.43	70.8	3.32<br>3.61	50.8	3.36<br>1.94	exo<br>endo
9	20.8	1.16	18.5	1.16	20.9	1.20	18.2	1.11	
10	23.2	0.93	20.4	0.98	23.6	0.96	20.3	0.92	

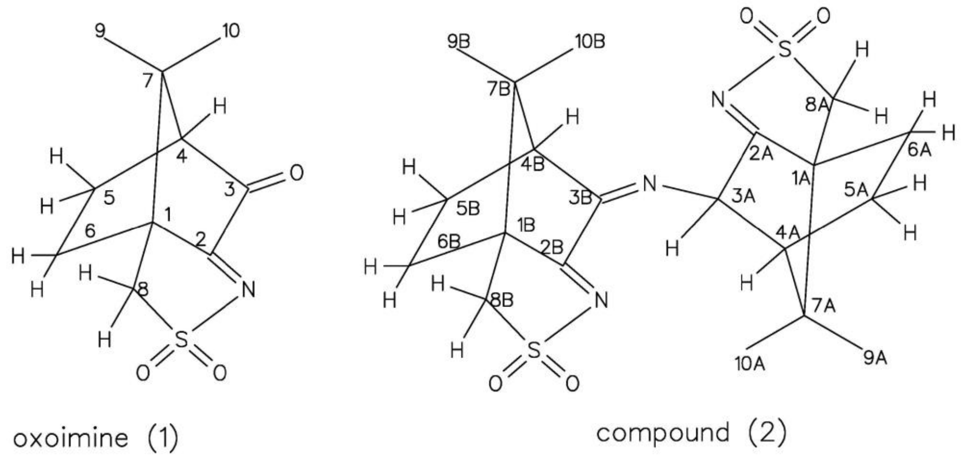

Figure S11: Numbering of the carbon atoms in compounds **1** and **2** used in the NMR tables. The hydrogen atoms carry the numbers of the carbon atoms to which they are directly attached. For compound **2**, the isomer with (3A*S*) configuration is shown.

The calculated isotropic chemical shifts were subtracted from the corresponding values for TMS in order to obtain the δ values familiar to the experimental list. These values were then plotted against the experimental values in the same solvent. The correlations thus obtained are shown in Figure S12.

The established correlations can now be applied as scaling equations to calculated chemical shifts of structurally similar compounds in order to obtain values which can directly be compared with experimental data. Similarly to the values in chloroform, we also calculated the corresponding scaling equations for acetonitrile as solvent. The scaling procedure is as follows: The calculated isotropic chemical shifts (δ_{calc}) of a compound (such as **2**) are referenced to TMS in the same solvent. Then, the scaling equations given below are applied in order to obtain the scaled values

(δ_{scal}). These can directly be compared with the experimental values of the chemical shifts in the same solvent and should be very similar, provided that the real structure is that which has been used for the calculations, and the solvent has been adequately treated.

For $CDCl_3$:

^{13}C: $\delta_{scal} = 0.9457 * \delta_{calc} - 5.070$ $R^2 = 0.9973$

^{1}H: $\delta_{scal} = 0.9794 * \delta_{calc} + 0.0440$ $R^2 = 0.9949$

For CD_3CN:

^{13}C: $\delta_{scal} = 0.9447 * \delta calc - 3.7411$ $R^2 = 0.9989$

^{1}H: $\delta_{scal} = 0.9989 * \delta calc - 0.0485$ $R^2 = 0.9911$

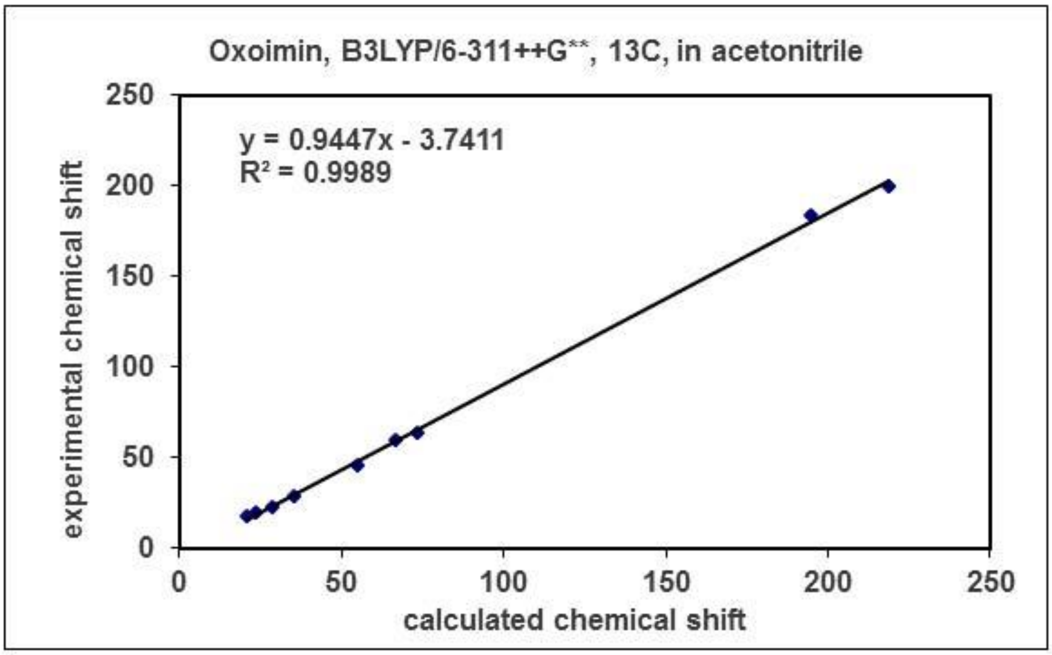

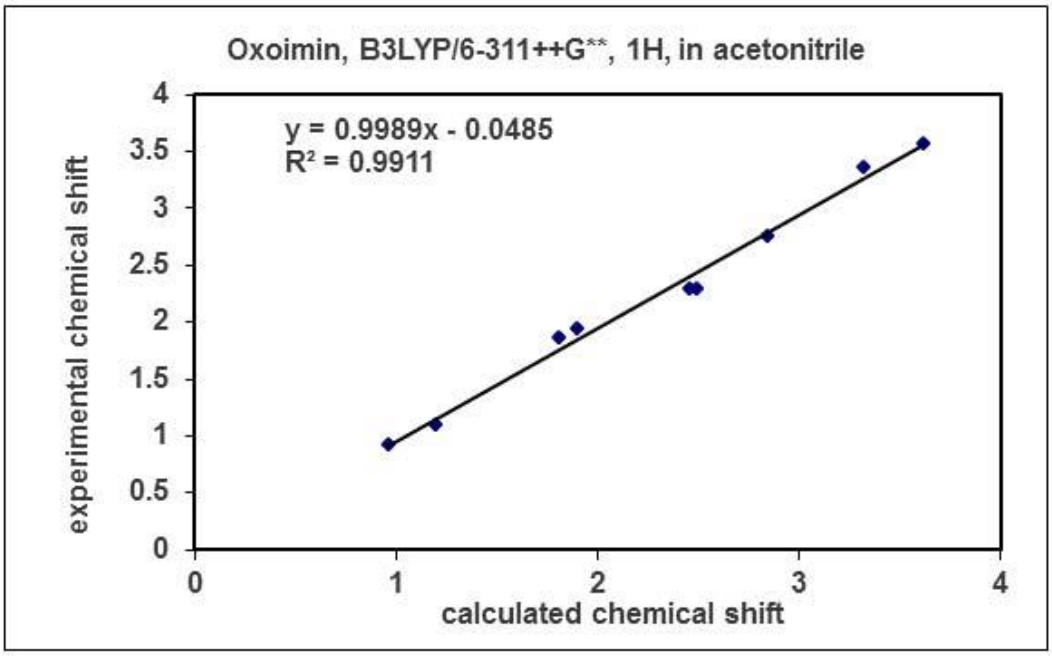

Figure S12: Correlation of calculated and experimental ^{1}H and ^{13}C NMR chemical shifts for oxoimine **1**. The R^2 values above 0.99 demonstrate the reliability of the equations.

2. Assignment of the configuration at carbon atom 3A in compound (**2**)

Compound **2** (see Figure S11) is the principal product of the reaction between the oxoimine **1** and amino acids (glycine, alanine, phenylalanine, leucine). The overall stereochemistry is determined by the starting material (1*S*) which is preserved in both camphor-derived subunits. In subunit A, a new center of chirality (3A) is created during the reaction, and its configuration is not a priori obvious. Based mainly on NOE measurements, the configuration (3A*S*) was assigned in compound **2**. We set out to confirm this assignment by chemical shift calculations. For this purpose, both diasteroisomers (having (3A*S*) and (3A*R*) configuration) were optimized with inclusion of the solvent acetonitrile (Cosmo method), and their ^{1}H and ^{13}C chemical shifts calculated and scaled

according to the equations given above. The comparison with the experimental values is shown in Table S2. The correlation of the ^{13}C data (Figure S13) is good for both isomers and does not allow for a decision between them. However, the corresponding ^{1}H correlations (Figure S14) show a much better fit for the (3A*S*) isomer, thus confirming the assignment based on experimental NMR, in particular NOE effects.

Table S2: Comparison of scaled calculated chemical shifts (δ_{scal}) of the two diastereoisomers of compound **2** with the experimental spectra in acetonitrile at −20 °C.

carbon	(3AS)		(3AR)		experimental		H stereochem.
1A	66.9	—	65.6	—	65.3	—	
2A	191.5	—	191.7	—	194.8	—	
3A	64.6	5.00	67.7	4.58	64.4	4.81	
4A	53.2	2.39	53.2	2.50	51.9	2.43	
5A	19.7	2.01	24.7	2.28	19.4	1.08	exo
		2.69		1.55		2.07	endo
6A	30.5	2.34	29.7	2.29	29.5	2.22	exo
		1.63		1.74		1.61	endo
7A	50.3	—	52.8	—	47.3	—	
8A	59.8	3.14	58.9	3.12	50.7	3.13	exo
		3.41		3.37		3.42	endo
9A	17.4	1.19	18.3	1.15	18.5	1.11	
10A	16.5	0.94	17.9	0.90	18.9	0.95	
1B	65.6	—	65.5	—	63.9	—	
2B	183.9	—	184.3	—	182.0	—	
3B	173.9	—	173.0	—	176.6	—	
4B	54.2	3.21	54.4	3.51	53.3	3.28	
5B	25.8	2.41	23.8	2.27	24.8	1.78	exo
		2.21		1.61		2.22	endo
6B	29.3	2.33	29.4	2.32	28.6	2.22	exo
		1.78		1.80		1.78	endo
7B	50.0	—	50.1	—	46.7	—	
8B	59.0	3.25	59.2	3.23	49.9	3.27	exo
		3.48		3.48		3.51	endo
9B	15.9	1.15	16.1	1.15	19.5	1.07	
10B	17.9	0.85	19.2	0.90	17.8	0.83	

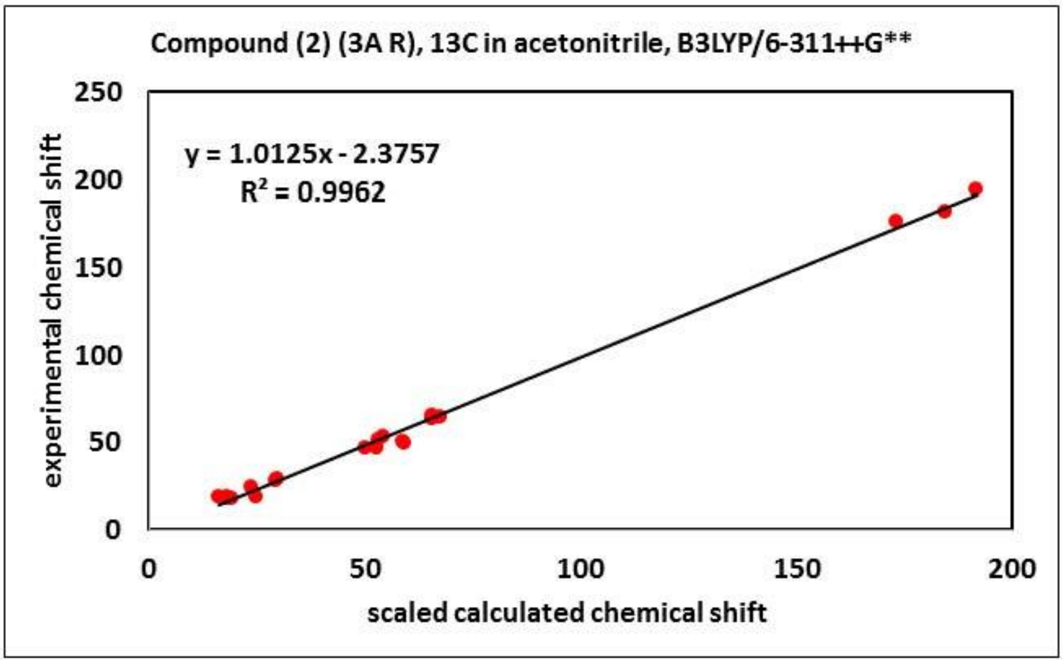

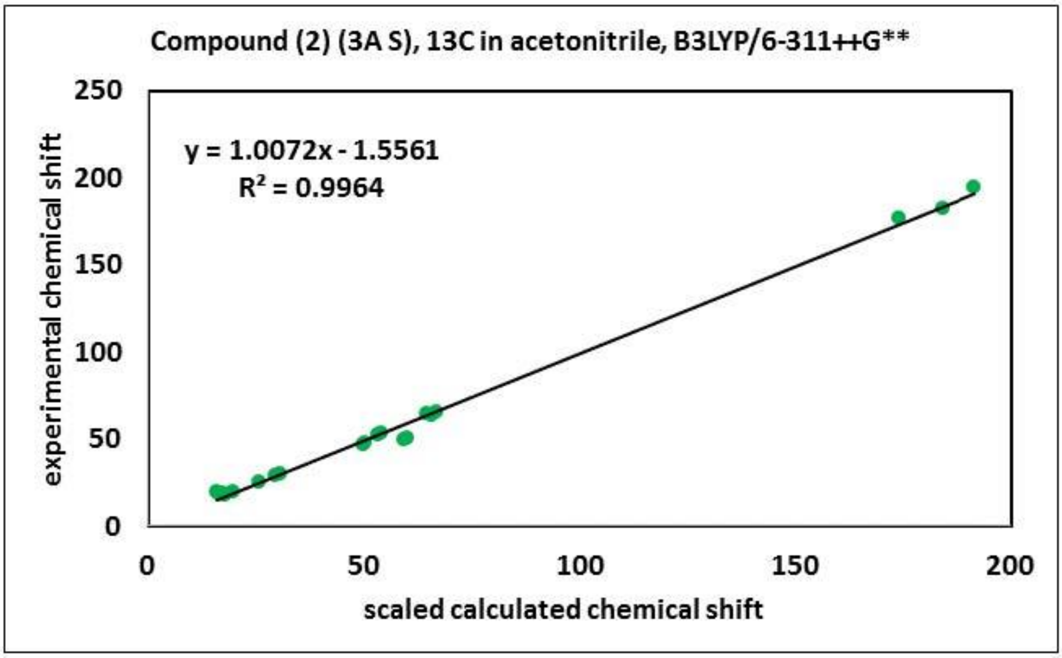

Figure S13: Correlation of scaled calculated and experimental ^{13}C NMR chemical shifts for the diastereoisomers of compound **2**. Both show a good correlation with almost equal R^2 values.

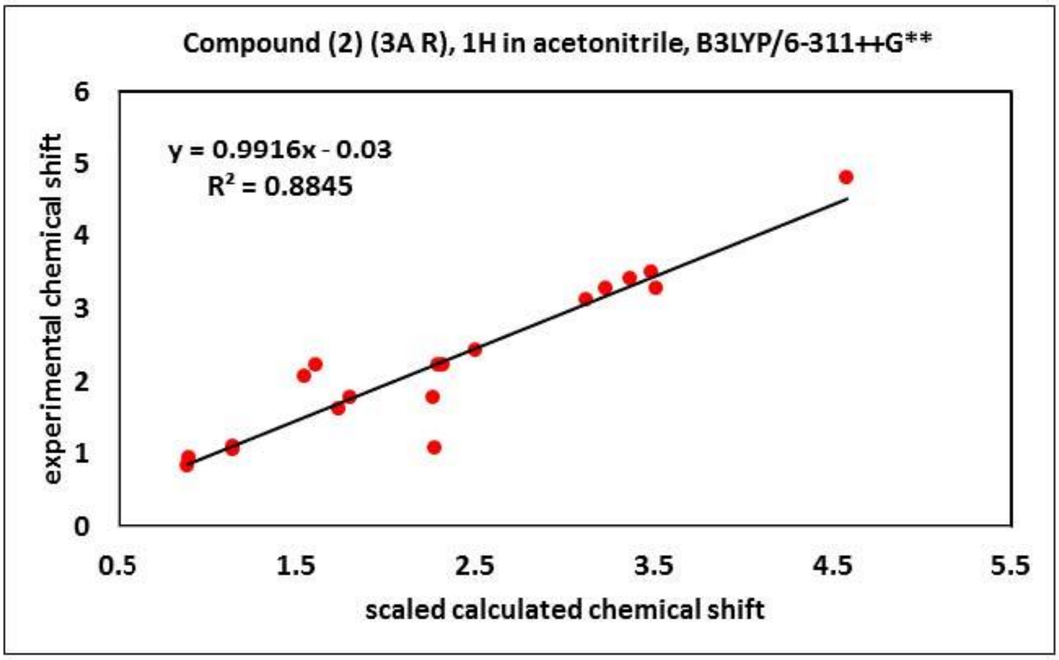

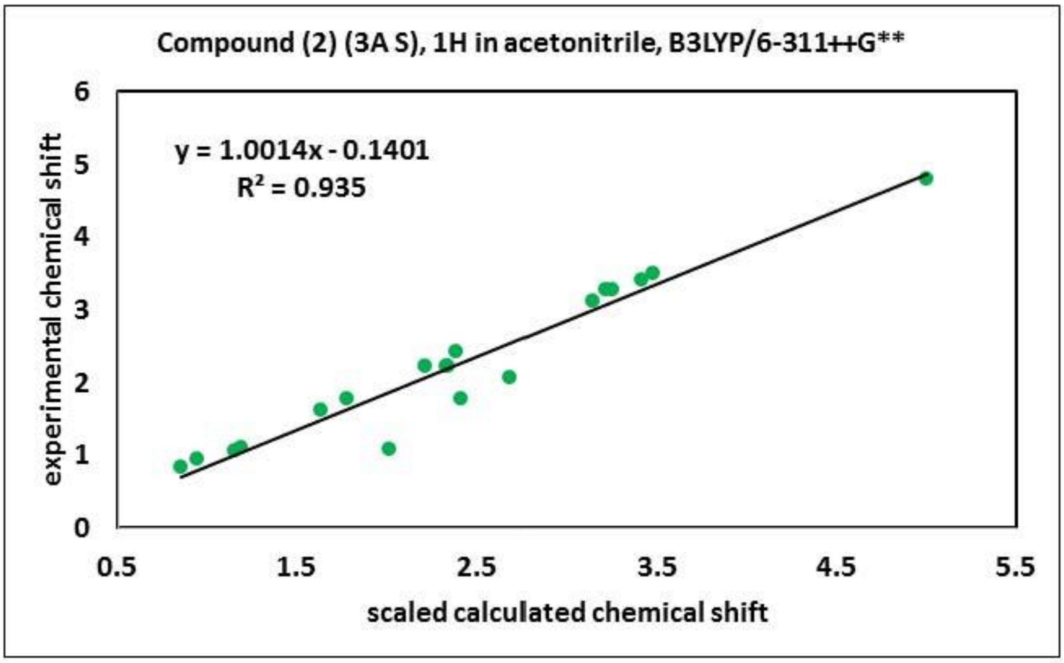

Figure S14: Correlation of scaled calculated and experimental ^{1}H NMR chemical shifts for compound **2** for isomers having *R* configuration at carbon atom 3A (above) and *S* (below). The better correlation for the *S* configuration supports the assignment based on coupling and NOE.

References

[1] Granovsky, A.A. PCGamess version7.1,http://classic.chem.msu.su/gran/gamess/

[2] Schmidt, M.W.; Baldridge, K.K.; Boatz, J.A.; Elbert, S.T.; Gordon, M.S.; Jensen, J.J.; Koseki, S.; Matsunaga, N.; Nguyen, K.A.; Su, S.; Windus, T.L.; Dupuis, M.; Montgomery Jr., J.A.; *J. Comput. Chem*. **1993**, *14*, 1347-1363.

[3] Valiev, M.; Bylaska, E.J.; Govind, N.; Kowalski, K.; Straatsma, T.P.; van Dam, H.J.J.; Wang, D.; Nieplocha, J.; Apra, E.; Windus, T.L.; de Jong, W.A. *Comp. Phys. Commun*. **2010**, *181*, 1477-1489.

[4] Schreckenbach, G.; Ziegler, T. *J. Phys. Chem*. **1995**, *99*, 606-611.

[5] Klamt, A.; Schüürmann, G. *J. Chem. Soc., Perkin Trans. 2*, **1993**, 799-805.

[6] Schaftenaar, G.; Noordik, J.H. J. Comput.-Aided Mol. Des. **2000**, *14*, 123-334.

[7] Glahsl, G.; Herrmann, R. *J. Chem. Soc., Perkin Trans. 1* **1988**, 1753-1757.

[8] Barone, G.; Gomez-Paloma, L.; Duca, D.; Silvestri, A.; Rico, R.; Bifulco, G. *Chem. Eur. J*. **2002**, *8*, 3233-3239.

[9] Lampropoulou, M.; Herrmann, R.; Wagner, G. *Tetrahedron* **2004**, *60*, 4635-4643.

[10] Smith, S.G.; Goodman, J.M. *J. Org. Chem*. **2009**, *74*, 4597-4607.

[11] Forsyth, D.A.; Sebag, A.B. *J. Am. Chem. Soc*. **1997**, *119*, 9483-9494.

[12] Jain, R.; Bally, T.; Rablen, P.R. *J. Org. Chem*. **2009**, *74*, 4017-4023.

[13] Bagno, A.; Rastelli, F.; Saielli, G. *Chem. Eur. J*. **2006**, *12*, 5514-5525.

[14] Dybiec, K.; Gryff-Keller, A. *Magn. Res. Chem*. **2009**, *47*, 63-66.

[15] Smith, S.G.; Goodman, J.M. *J. Am. Chem. Soc*. **2010**, *132*, 12946-12969.

[16] Cimino, P.; Gomez-Paloma, L.; Duca, D.; Riccio, R.; Bifulco, G. *Magn. Res. Chem*. **2004**, *42*, S26-S33.

[17] Dokalik, A.; Kalchhauser, H.; Mikenda, W.; Schweng, G. *Magn. Res. Chem*. **1999**, *37*, 895-902.

[18] Wiitala, K.W.; Cramer, C.J.; Hoye, T.R. *Magn. Res. Chem*. **2007**, *45*, 819-829.

[19] Swart, M.; Solà, M.; Bickelhaupt, F.M. *J. Chem. Phys*. **2009**, *131*, 094103.

[20] Swart, M.; Solà, M.; Bickelhaupt, F.M. *J. Comp. Meth. Sci. Engin*. **2009**, *9*, 69-77.
