# Supplementary material for: Strecker degradation of amino acids promoted by a camphor-derived sulfonamide
Source: Beilstein J Org Chem. 2016 Apr 18;12:732–44. doi: 10.3762/bjoc.12.73 (PMC4902088; doi:10.3762/bjoc.12.73)
Supplement: File 4 — Calculated reaction (IRC path) via the transition state 6g → 11. [file Beilstein_J_Org_Chem-12-732-s004.pdf]

Supporting Information File 3

for

Strecker degradation of amino acids promoted by a camphor-derived sulfonamide

M. Fernanda N. N. Carvalho[1]*, M. João Ferreira[1], Ana S. O. Knittel[1], Maria da Conceição Oliveira[1], João Costa Pessoa[1], Rudolf Herrmann[2], and Gabriele Wagner[3]

Address: [1]Centro de Química Estrutural, Instituto Superior Técnico, Universidade de Lisboa, Av. Rovisco Pais 1049-001 Lisboa, Portugal, [2]Institut für Physik, Universität Augsburg, Universitätsstr. 2, 86159 Augsburg, Germany and [3]University of Chester, Faculty of Science and Engineering, Department of Natural Sciences, Thornton Science Park, Pool Lane, Ince, Chester, CH2 4NU, UK

Email: Maria Fernanda N. N. Carvalho - fcarvalho@ist.utl.pt

*Corresponding author

Calculations for Strecker degradation via zwitterions in the case of glyoxal and ninhydrin

Calculations

Decarboxylation of amino acid imines with diketones and similar compounds — The Strecker degradation

Geometry optimizations, Hessian calculations, saddle point searches and intrinsic reaction path (IRC) calculations [1,2,3] were done with the program PCGAMESS (version 7.1) [4,5], using B3LYP/6-31G**, or B3LYP/6-31++G** for anions. Zero point energies and basis set superposition errors were not included since no high precision numerical results were needed. Data analysis and visualization was done with MOLDEN [6].

α-Amino acids are the basis of life on earth and are compounds of remarkable chemical stability. But when they are treated with compounds containing carbonyl groups, they show a pronounced tendency towards loss of CO_2. This reaction was first observed by Strecker in 1862 [7] and is therefore referred to as Strecker degradation. It was found to be responsible for flavor development during heating of food [8]. Since this reaction is also part of the sequence leading to the formation of compound **2** from the oxoimine **1** by the reaction with α-amino acids, we decided to explore the mechanism of the decarboxylation reaction in more detail. In almost all mechanistic investigations, the loss of CO_2 is taken "as granted" and not further questioned, and the focus lies on tautomerization reactions (H migrations) occurring later in the sequence. We present here results concerning the prototypic reaction of glyoxal, the simplest compound which shows this behavior, and the case of ninhydrin since it is of great importance in analytics. The optimized structures of the imines of these compounds with glycine are shown in Figure S15.

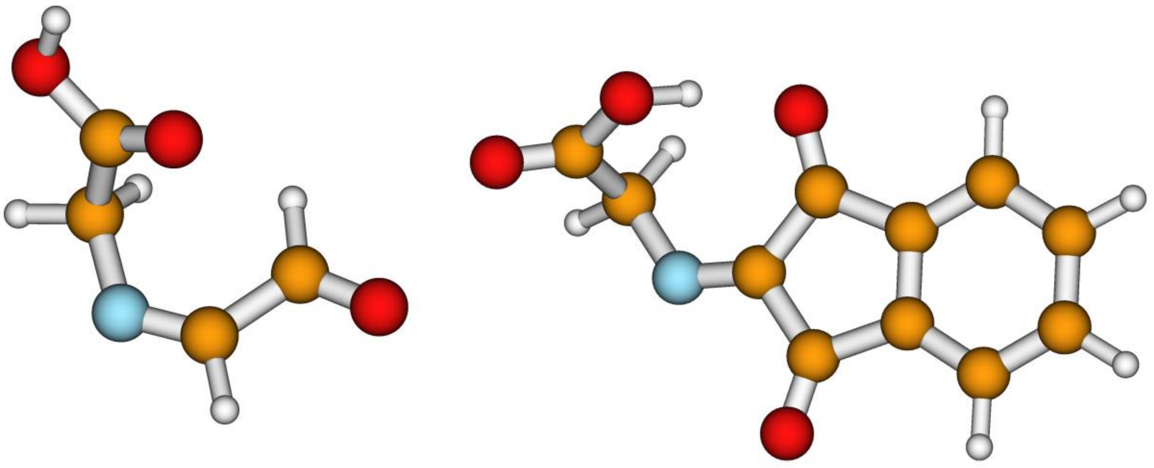

Figure S15: Optimized structures of glycine imines with glyoxal and ninhydrin.

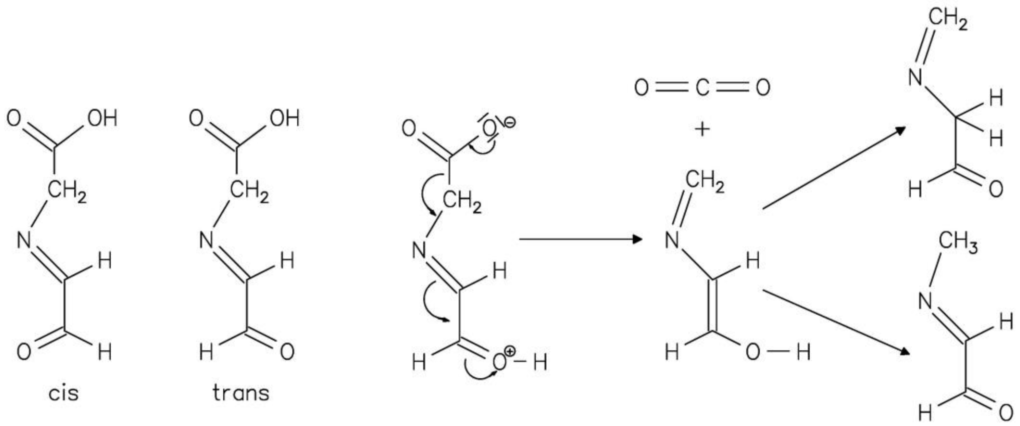

Figure S16: *Cis* and *trans* conformers of the imine of glycine with glyoxal, and degradation of the zwitterion to form CO_2 and typical tautomerization products.

1. The minimal example (glyoxal)

Summarizing the experimental evidence for the Strecker degradation of α-amino acids, it was concluded that efficient promoters are of the type O=C(R^1)–(CH=CH)$_n$-C(R^2)=O where n = 0,1 ... [9,10], and one of the carbonyl groups (C=O) may be replaced by an imine (C=NR) without loss of reactivity.

Taking the imine of glycine with glyoxal as the simplest case ($R^1 = R^2$ = H, n = 0), we can calculate two stable isomeric structures (conformers) which we denote as cis and trans (see Figure S16; Figure S15 shows the *trans* conformer). They do not differ significantly in energy (the trans structure is only by 1.3 kcal/mol more stable) and can interconvert by rotation around the central HC–CH single bond. Removing the carboxylic proton to form the anion does not impair the stability of the compound, nor does protonation at either the nitrogen atom or the carbonyl oxygen to form the cation. The situation changes dramatically when the zwitterion is generated by removing the proton of the carboxyl group and simultaneously protonating the carbonyl oxygen. Geometry optimization immediately leads to CO_2 loss, without any noticeable barrier. The probable electron flow is shown in Figure S16, together with typical Strecker products after tautomerization. In aqueous media these imines will finally be hydrolyzed to the aldehydes and amines.

We have studied a potential intramolecular pathway from the starting *cis*-glyoxalimine to the decarboxylated product by the intrinsic reaction coordinate (IRC) method [1]. We found that proton transfer from the carboxylic group to the carbonyl oxygen and bond breaking to form CO_2 occur almost simultaneously close to the transition state (TS) (see Figure S18). The calculated activation energy (as difference between the TS and the minimum energy trans-imine) is $\Delta E^{\ddagger}$= 24.2 kcal/mol, and is to be seen rather as an upper limit since in the real reaction water molecules can promote the proton transfer. The overall reaction is exothermic (calculated ΔE = −5.1 kcal/mol). In practice, CO_2 evolution from the reaction mixture makes the reaction irreversible.

The total path along the intrinsic reaction coordinate (IRC) can be seen as movie (Supporting Information File 8), and the change in energy and salient distances in Figure S17.

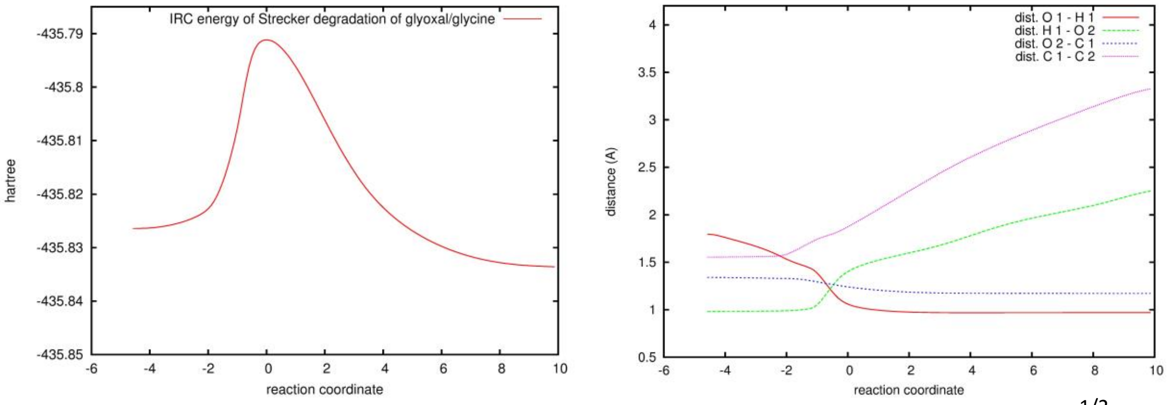

Figure S17: IRC path along the mass-weighted reaction coordinate (values in amu$^{1/2}$*bohr). For numbering of the atoms, see Figure S18 (right).

To better understand the TS structure we have undertaken an Atom-In-Molecules (AIM) analysis [11,12]. The most important results are given in Table S3 and are shown graphically in Figure S18 (middle and right). All atoms involved directly in the reaction are connected by bond paths[1]. The electron density ρ at the bond critical points (bcp, shown in Figure S18 (right) where basin borders (green) and bond paths (blue) intersect) is in all cases still higher than typical values for hydrogen bonds (~ 0.13 e/Å³) [12]. This means that the bond between H1 and the carbonyl oxygen O1 is already formed, but bond breaking occurs only after passage of the TS. This is in accord with the pronounced changes in bond lengths shortly before the TS in the IRC path where bond formation occurs and bond breaking is prepared (Figure S17).

Table S3: Results of the AIM analysis of the transition state at the bond critical points. Only critical points (cp) between the atoms participating in bond breaking / formation are given. ρ Denotes the electron density, $\nabla^2\rho$ its Laplacian, and ε the ellipticity (the low values indicate that there is no significant π-bonding involved). For the numbering of the atoms, see Figure S18 (right).

atom A	atom B	r(A-cp) (Å)	r (B-cp) (Å)	ρ (e/Å^3)	$\nabla^2\rho$ (e/Å^5)	ε
O 1	H 1	0.849	0.210	1.778	-36.65	0.011
H 1	O 2	0.400	1.002	0.655	3.404	0.024
O 2	C 1	0.810	0.431	2.584	-9.787	0.032
C 1	C 2	0.917	0.957	0.749	-1.116	0.027

[1] For convenience, we use the expression “bond path” to indicate the lines of maximal electron density connecting nuclei also for non-equilibrium structures instead of “atomic interaction line”.

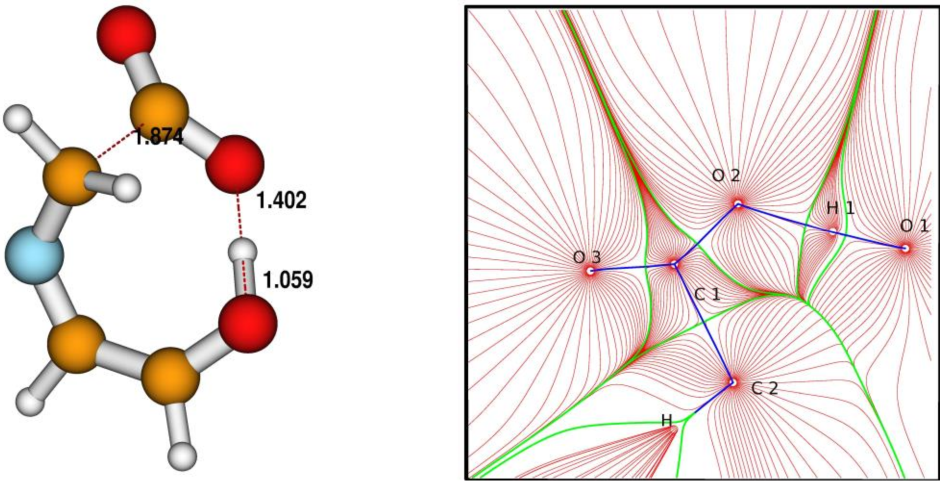

Figure S18: Left: Calculated transition-state structure for the reaction of the cis conformer of the imine of glycine with glyoxal via intramolecular proton transfer. The proton transfer is at an intermediate stage and the $H_2C–CO_2$ single bond is considerably elongated. Salient distances are given on the plot (Å). Right: Contour plot of the gradient $\nabla\rho$ vector paths (red) in the plane containing the atoms which participate in the bond cleavage/bond formation steps. The vector paths start at a nucleus and follow the steepest descent of the electron density. The borders of the zero flux basins of the atoms are shown in green and the bond paths in blue, including atom numbering used in Table S3. The atoms are connected, i.e. there is appreciable electron density at the bond critical points.

In contrast to the outlined mechanism of the Strecker degradation via zwitterions, several authors have suggested that the initially formed imines of amino acids should give oxazolidin-5-ones as stable intermediates, from which azomethine ylides (with hydrogen at the nitrogen atom) are formed by a cycloreversion with loss of CO_2 [13-20]. We have therefore calculated the ring opening of the potential intermediate formed from glyoxal and glycine, 2-formyl-oxazolidin-5-one (see Figure S19). The overall reaction turned out to be endothermic (8.0 kcal/mol). Indeed, all reported experimental procedures require heating (melt or reflux) for the decomposition of the corresponding oxazolidinones to occur, and are possible only because of the removal of gaseous CO_2 which drives the reaction to completion. The activation energy was calculated to be 24.7 kcal/mol, which is similar to that discussed above for the exothermic intramolecular reaction. In contrast to the mechanism via zwitterions, the second carbonyl group of the 1,2-dicarbonyl compound is only a spectator substituent and does not promote the cycloreversion. We therefore think that the formation of oxazolidin-5-ones is in principle possible, but if they can act as intermediates or remain as side products is mainly a question of the reaction conditions; without increase of temperature the Strecker degradation will probably not occur.

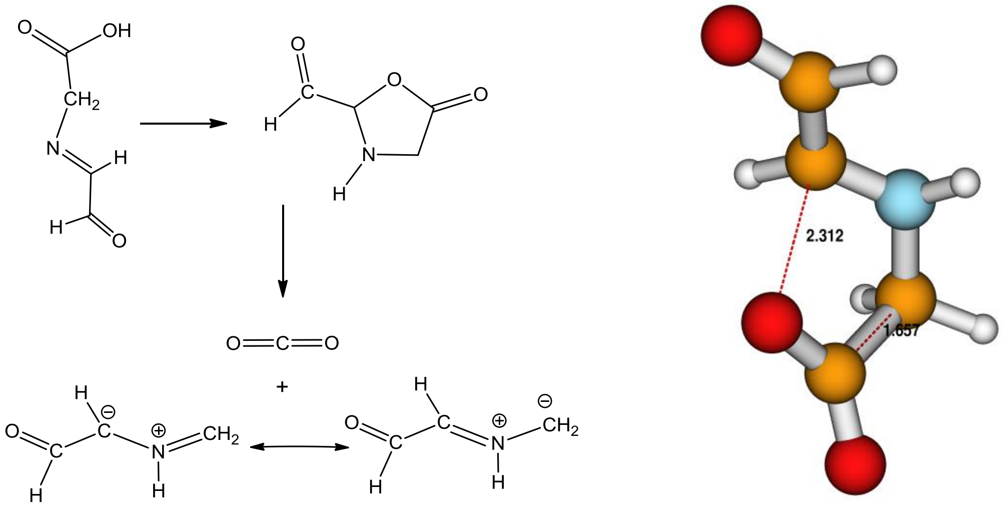

Figure S19: Left: Cycloreversion of 2-formyl-oxazolidin-5-one to form the azomethine ylide; right: transition state of this reaction.

2. The case of ninhydrin

The reaction of ninhydrin (Figure S20) with amino acids to form a highly colored dimeric compound known as Ruhemann's purple is of great importance in the analytics of amino acids. It is one of the best studied examples of a reaction sequence having a Strecker degradation as key step [21]. As the focus of most mechanistic investigations was on the tautomerization reactions following the decarboxylation step [22,23], and for the proper Strecker degradation, suggestions on the decarboxylation pathway are rare [21,24,13]. The intense color of Ruhemann's purple is due to a mesomeric anionic structure formed by deprotonation of the compound shown in Figure S20 or its tautomers [25,26].

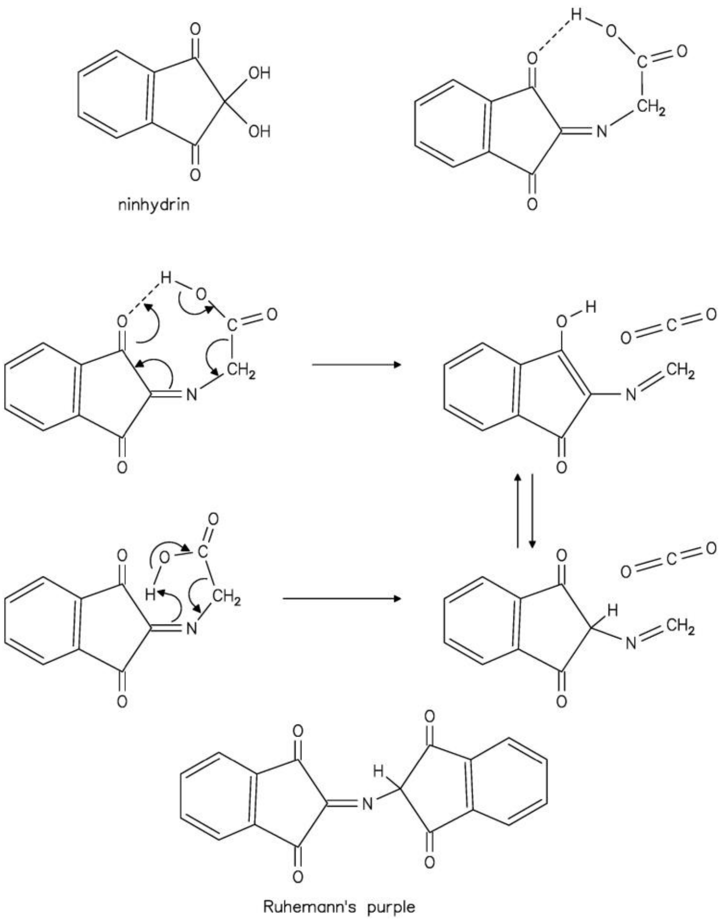

Figure S20: First row: ninhydrin and its imine with glycine (showing the numbering of the atoms participating in the Strecker degradation). Second row: a probable mechanism for the decarboxylation. Third row: textbook mechanism for the decarboxylation [27,28]. Fourth row: tautomer of Ruhemann's purple analogous to compound **2**.

Applying the results obtained with glyoxal to ninhydrin, we expected that the formation of zwitterionic structures of the imine with amino acids might be of importance as well. We again studied the behavior of the imine with glycine and found that the zwitterion formed by deprotonation of the carboxyl group and protonating one of the neighboring carbonyl groups of the ninhydrin moiety behaves exactly analogous to what was observed with glyoxal. In addition, we found that the zwitterion resulting from protonation at the imine nitrogen is not stable and loses CO_2 upon geometry optimization. This mechanism was proposed as a route to azomethine ylides (with hydrogen as substituent at the nitrogen atom) which were subsequently trapped as 1,3-dipolar cycloadducts [13]. Our results (see Supporting Information File 9 for this step) show that the postulated mechanism via azomethine ylides should indeed be viable under mild reaction conditions. In addition to the mechanism with zwitterions, we found that the mechanistic possibilities of ninhydrin imines are increased. In contrast to the glyoxal imine, where deprotonation of the carboxyl group alone does not impair the stability of the anion, we observed that with the imine of glycine and ninhydrin, easy decarboxylation of the anion occurs upon geometry optimization, in total analogy to the zwitterionic structure. We interpret this

difference as due to the presence of two additional carbonyl groups in the imine as compared to only one in the glyoxal imine, which allows for better delocalization and thus stabilization of the negative charge. Anionic intermediates in the ninhydrin reaction have been suggested earlier [23,24], and our results support this possibility.

Since CO_2 loss by geometry optimization of zwitterions and anions appears to occur without detectable barrier, there must be an energetic obstacle in the formation of the zwitterion or in the deprotonation step to form the anion. As for the imine with glyoxal, we therefore calculated an intramolecular pathway for forming the zwitterion by hydrogen transfer from the carboxylic group to one of the carbonyl oxygen atoms (Figure S20, second row). The transition-state structure is compared with that of the starting imine in Figure S21, and energy and salient distances along the intrinsic reaction path are shown in Figure S22. A movie along the IRC pathway is also included in Supporting Information File 10. For the analogous reaction with a direct transfer of a proton to the imine carbon atom (Figure S20, third row), which appears in textbooks [27] and online-databases [28], we could not locate a transition state nor find any other indication that the reaction might be feasible.

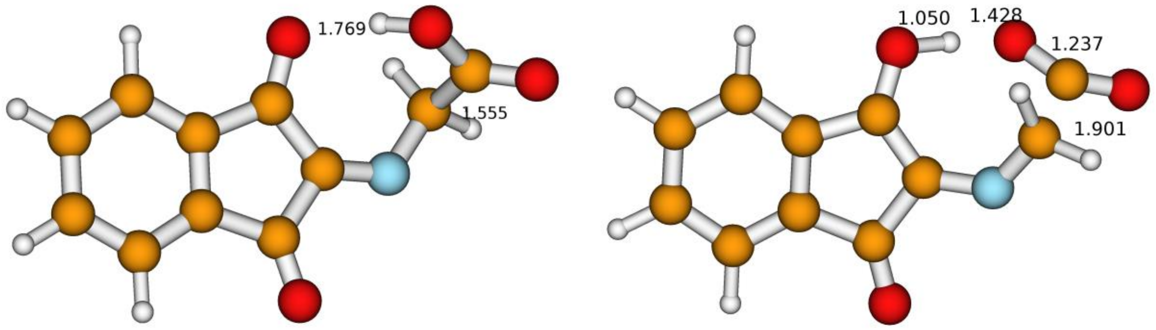

Figure S21: Left: equilibrium geometry of the imine of glycine with ninhydrin. Right: transition-state geometry for the intramolecular Strecker degradation of this imine. The activation energy was calculated as $\Delta E^{\ddagger}$ = 21.9kcal/mol.

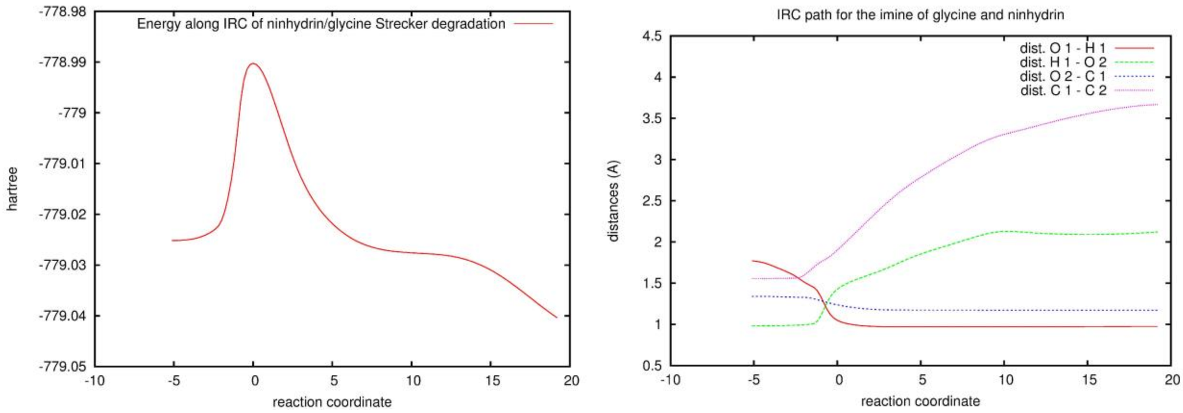

Figure S 22: IRC path along the mass-weighted reaction coordinate (values in $amu^{1/2}$*bohr). The

overall reaction is exothermic (Δ*E* = −13.9kcal/mol). For numbering of the atoms, see Figure S20.

The tautomeric products (Figure S23) of the decarboxylation reaction are of rather similar energy, and under the slightly acidic reaction conditions (pH~5 [22]) we can expect an equilibrium between all of them. The azomethine ylide **D** is surprisingly stable, and this may explain why products of its 1,3-dipolar cycloaddition with maleimide could be trapped during the course of the ninhydrin reaction with amino acids [13].

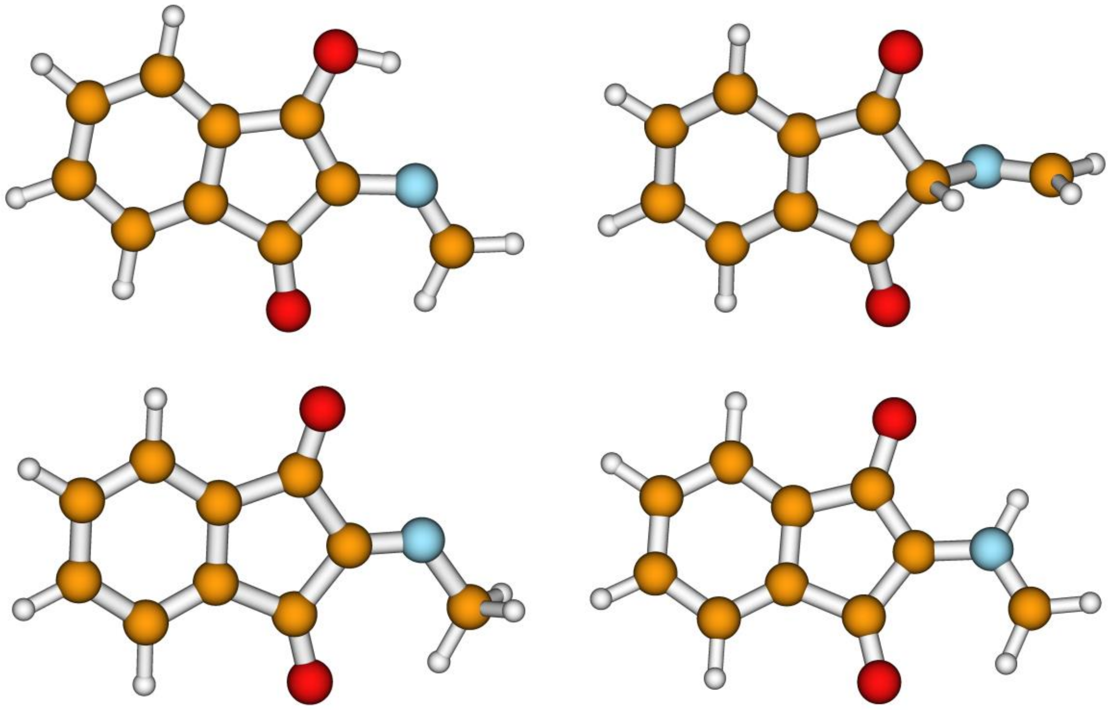

Figure S23: Tautomeric products expected after the decarboxylation reaction. Upper left: tautomer **A** as expected from the proposed reaction mechanism; Δ*E* = 2.2kcal/mol. Upper right: tautomer **B**; Δ*E* = 4.3kcal/mol. Lower left: tautomer **C**; Δ*E* = 0.6kcal/mol. Lower right: tautomer **D**, azomethine ylide, most stable isomer, Δ*E* = 0.

An aminal (carbinolamine) structure (where one of the hydroxy groups of ninhydrin is substituted by the amino acid) has been proposed as starting point for a simultaneous dehydration and decarboxylation [21]. However, we could not find any evidence that this might be a feasible mechanism. The aminal is stable as such (with a hydrogen bond between the carboxylic hydrogen and one of the carbonyl oxygen atoms), and any attempt to transfer the carboxylic hydrogen to the remaining hydroxy group of ninhydrin to preform a water molecule to be eliminated failed, since it was transferred back to its original position.

Conclusion: Imines of amino acids with carbonyl compounds have a tendency towards loss of CO_2 upon deprotonation of the carboxyl group. Without this negative charge at the carboxyl

group, decarboxylation is difficult. The deprotonation may occur either directly (by water molecules or other proton acceptors) or by an intramolecular proton transfer from the carboxylic acid to a carbonyl oxygen or a double-bonded nitrogen (imine). If a deprotonation is followed by a decarboxylation or not, depends on the ability of the thus formed molecule to accommodate the negative charge. For zwitterionic structures, as in the imines of 1,2-dicarbonyl compounds, this is easily achieved by the positive charge at a protonated C=O or C=N double bond (including the imine nitrogen of the amino acid component itself). Charge delocalization over "conducting" C=C bonds conserves this anion-stabilizing property and is the reason behind Schönberg's rule of the O=C–(CH=CH)*n*–C=O groups. Such compounds therefore form imines that undergo the Strecker degradation already at or slightly above room temperature. If the second C=O or C=N bond is missing, the formation of a zwitterion with a positive charge at the imine nitrogen of the original amino acid (azomethine ylide route) is the only possibility for a Strecker degradation but less efficient. This path generally needs higher temperature to proceed, as does the introduction of electron-accepting groups (as in nitrobenzaldehydes).
The mechanism presented here also explains why the Strecker degradation proceeds particularly well at pH~5 which is sufficiently acidic to catalyze the formation and hydrolysis of imines and in a suitable range where zwitterions can be expected to occur in reasonable concentrations.

References

[1] Ishida, U.; Morokuma, K.; Kormonicki, A.; *J. Chem. Phys*. **1977**, *66*, 2153-2156.

[2] Schmidt, M.W.; Gordon, M.S.; Dupuis, M. *J. Am. Chem. Soc*. **1985**, *107*, 2585-2589.

[3] Baldridge, K.K.; Gordon, M.S.; Steckler, R.; Truhlar, D.G.; *J. Phys. Chem*. **1989**, *93*, 5107-5119.

[4] Granovsky, A.A. PCGamess version7.1, http://classic.chem.msu.su/gran/gamess/

[5] Schmidt, M.W.; Baldridge, K.K.; Boatz, J.A.; Elbert, S.T.; Gordon, M.S.; Jensen, J.J.; Koseki, S.; Matsunaga, N.; Nguyen, K.A.; Su, S.; Windus, T.L.; Dupuis, M.; Montgomery Jr., J.A. *J. Comput. Chem*. **1993**, *14*, 1347-1363.

[6] Schaftenaar, G.; Noordik, J.H. *J. Comput.-Aided Mol. Des*. **2000**, *14*, 123-334.

[7] Strecker, A. *Liebigs Ann. Chem*. **1862**, *123*, 363-367.

[8] Yaylayan, V.A. *Food Sci. Technol. Res*. **2003**, *9*, 1-6.

[9] Schönberg, A.; Moubasher, R.; Mostafa, A. *J. Chem. Soc*. **1948**, 176-182.

[10] Schönberg, A.; Moubacher, R. *Chem. Rev*. **1952**, *50*, 261-277.

[11] Bader, R.W.F. *Theory of Atoms in Molecules*, http://www.chemistry.mcmaster.ca/aim/aim_0.html

[12] Bader, R.W.F.; Essén, H. *J. Chem. Phys*. **1984**, *80*, 1943-1960.

[13] Grigg, R.; Malone, J.F.; Mongkolaussavaratana, T.; Thianpatanagul, S. *Tetrahedron* **1989**, *45*, 3849-3862.

[14] Grigg, R.; Surendrakumar, S.; Thianpatanagul, S.; Vipond, D. *J. Chem. Soc., Perkin Trans. 1* **1988**, 2693-2701.

[15] Tsuge, O.; Kanemasa, S.; Ohe, M.; Takenaka, S. *Bull. Chem. Soc. Jpn*. **1987**, *60*, 4079-4089.
[16] Manini, P.; D'Ischia, M.; Prota, G. *J. Org. Chem*. **2001**, *66*, 5048-5053.
[17] Yaylayan, V.A.; Wnorowski, A.; Perez Locas, C.; *J. Agric. Food Chem*. **2003**, *51*, 1753-1757.
[18] Stadler, R.H.; Robert, F.; Riediker, S.; Varga, N.; Davidek, T.; Devaud, S.; Goldmann, T.; Hau, J.; Blank, I.; *J. Agric. Food Chem*. **2004**, *52*, 5550-5558.
[19] Perez Locas, C.; Yaylayan, V.A. *J. Agric. Food Chem*. **2008**, *56*, 6069-6074.
[20] Chu, F.L.; Yaylayan, V.A. *Carbohydr. Res*. **2009**, *344*, 229-236.
[21] McCaldin, D.J. *Chem.Rev*. **1960**, 39-51.
[22] Lamothe, P.J.; McCormick, P.G. *Analyt. Chem*. **1972**, *44*, 821-825.
[23] Friedman, M.; Williams, L.D. *Bioorg. Chem*. **1974**, *3*, 267-280.
[24] Bottom, C.B; Hanna, S.S.; Siehr, D.J. *Biochem. Educ*. **1978**, *6*, 4-5.
[25] Wigfield, D.C.; Buchanan, G.W.; Croteau, S.M. *Can. J. Chem*. **1980**, *58*, 201-205.
[26] Dietz, F.; Rommel-Möhle, K.; Schleitzer, A.; Tyutyulkov, N. *Z. Naturforsch. B* **1993**, *48*, 1133-1137.
[27] Vollhardt, K.P.C.; Schore, N.E. *Organic Chemistry*, 4th ed., W.H. Freeman and Co., New York **2003**, 1194-1195.
[28] http://www.ecompound.com/Reaction%20reference/name_reaction_alphabetical4.htm
